# Genetic disruption of *slc4a10* alters the capacity for cellular metabolism and vectorial ion transport in the choroid plexus epithelium

**DOI:** 10.1186/s12987-019-0162-5

**Published:** 2020-01-07

**Authors:** Inga Baasch Christensen, Qi Wu, Anders Solitander Bohlbro, Marianne Gerberg Skals, Helle Hasager Damkier, Christian Andreas Hübner, Robert Andrew Fenton, Jeppe Praetorius

**Affiliations:** Department of Biomedicine, Health, Aarhus University, Wilhelm Meyers Allé 3, r. 219, 8000 Aarhus C, Denmark

**Keywords:** Choroid plexus, Cerebrospinal fluid, Ncbe, Mass spectrometry

## Abstract

**Background:**

Genetic disruption of *slc4a10*, which encodes the sodium-dependent chloride/bicarbonate exchanger Ncbe, leads to a major decrease in Na^+^-dependent HCO_3_^−^ import into choroid plexus epithelial cells in mice and to a marked reduction in brain intraventricular fluid volume. This suggests that Ncbe functionally is a key element in vectorial Na^+^ transport and thereby for cerebrospinal fluid secretion in the choroid plexus. However, *slc4a10* disruption results in severe changes in expression of Na^+^,K^+^-ATPase complexes and other major transport proteins, indicating that profound cellular changes accompany the genetic manipulation.

**Methods:**

A tandem mass tag labeling strategy was chosen for quantitative mass spectrometry. Alterations in the broader patterns of protein expression in the choroid plexus in response to genetic disruption of Ncbe was validated by semi-quantitative immunoblotting, immunohistochemistry and morphometry.

**Results:**

The abundance of 601 proteins were found significantly altered in the choroid plexus from Ncbe ko mice relative to Ncbe wt. In addition to a variety of transport proteins, particularly large changes in the abundance of proteins involved in cellular energy metabolism were detected in the Ncbe ko mice. In general, the abundance of rate limiting glycolytic enzymes and several mitochondrial enzymes were reduced following *slc4a10* disruption. Surprisingly, this was accompanied by increased ATP levels in choroid plexus cells, indicating that the reduction in capacity for energy metabolism was adaptive to high ATP rather than causal for a decreased capacity for ion and water transport. Ncbe-deficient cells also had a reduced cell area and decreased K^+^ content.

**Conclusion:**

Our findings suggest that the lack of effective Na^+^-entry into the epithelial cells of the choroid plexus leads to a profound change in the cellular phenotype, shifting from a high-rate secretory function towards a more dormant state; similar to what is observed during ageing or Alzheimer’s disease.

## Background

A large fraction of the cerebrospinal fluid (CSF) is produced by the choroid plexus (CP) in the brain ventricles [1]. At this site, the choroid plexus epithelial cells (CPECs) secrete solutes and water at very high rates that is not surpassed by other mammalian epithelia [2]. The mechanisms for water and salt secretion are still debated i.e. the nature of the Na^+^ entry and exit mechanisms, as well as the transepithelial water transport pathways. The luminal Na^+^,K^+^-ATPase is the driving mechanism for solute secretion both through its direct extrusion of Na^+^ into the CSF and by creation of the driving force for secondary active transport processes [3–5]. The canonical water channel, aquaporin 1 (AQP1) is abundant in the luminal membrane of CPECs and is the major water transport pathway in the CP [6]. As the major HCO_3_^−^ exit pathway, electrogenic Na^+^:HCO_3_^−^ transport by NBCe2 most likely contributes to the Na^+^ extrusion by CPECs, while the direction of transport for the Na^+^,K^+^,2Cl^−^ cotransporter 1 (NKCC1) and the possible cotransport of water are matters of current discussion [7, 8]. The *slc4a10* gene product, Ncbe, is a Na^+^:HCO_3_^−^ import protein abundantly expressed in the basolateral membrane of CPECs, which in rodents couples ion import to Cl^−^ extrusion [9–11]. Genetic disruption of *slc4a10* leads to an approximately 80% decrease in brain ventricle volume mirrored by a cellular Na^+^ dependent HCO_3_^−^ import. Thus, we have proposed Ncbe as a main candidate for the Na^+^ entry mechanism [12].

Targeted, antibody-based studies of *slc4a10* knockout mice (Ncbe ko) relative to wildtype (Ncbe wt) controls, uncovered several changes in the expression of key transporters in CPECs. Notably, two of the most prominent luminal membrane proteins in CSF secretion were affected by the genetic *slc4a10* disruption; we found reduced expression levels of the Na^+^,K^+^-ATPase and the AQP1 to approximately 20% in the Ncbe ko mice, as compared to Ncbe wt mice [13]. The CPECs also displayed inversed localization of the Na^+^/H^+^ exchanger NHE1 from the luminal side to the basolateral side in Ncbe ko [14] and further changes in cell polarity, cell contact and anchoring proteins [15]. Many of these changes would be consistent with a reduction of cellular secretory capacity.

Based on these studies, we hypothesized that this very efficient transporting epithelium adapts to the lack of a major ion transporter by shutting down a secretory program and develops into a more dormant epithelial phenotype. To examine this in detail, we initially used a semi-quantitative proteomic approach to comprehensively compare CPECs from Ncbe ko and wt mice. This would delineate the cellular consequences of *slc4a10* disruption and generate new hypothesis on cellular adaptations to the loss of a major Na^+^ uptake mechanism. Immunoblotting, semi-quantitative immunofluorescence histochemistry, measurements of cellular ATP and K^+^ contents, and cell size estimations were also applied to determine additional changes in functional capacities of CPECs evoked by genetic *slc4a10* disruption. The major findings are that disruption of *slc4a10* alters the capacity for cellular metabolism and vectorial ion transport in the CP epithelium.

## Materials and methods

### Animals

Heterozygous breeding and genotyping of the *slc4a10*-targeted knockout mouse model have previously been described [12]. The mice were bred on c57bl/6 (Taconic) background, and both female and male mice aging 4–5 weeks were used. All procedures conformed to Danish animal welfare regulations. The authors are licensed to breed the mouse strain by The Animal Experiments Inspectorate, Ministry of Food, Agriculture, and Fisheries of Denmark (j.n. 2012-15-2935-00004).

### Tandem mass tag 10-plex isobaric mass tag labelling

Mice were euthanized under isoflurane anaesthesia, and CP tissues from all four ventricles of each mouse were isolated in 4 °C phosphate buffered salt solution (isotonic PBS, in mM: 167 Na^+^, 150.0 Cl^−^, 2.8 H_2_PO_4_^−^, 7.2 HPO_4_^2−^, pH 7.4), before the tissues were snap-frozen in liquid nitrogen. The tissues were thawed on ice and 5% Na-deoxycholate in 50 mM NH_4_HCO_3_ with protease inhibitors (Halt™ Protease Inhibitor Cocktail, Thermo Scientific) was added before sonication and centrifugation at 16,000×*g* for 10 min at 4 °C. The resulting supernatants were concentrated on spin columns (Amicon^®^ Ultra 0.5 mL, 3 kDa cut-off, 14,000×*g*). The samples were reduced, alkylated and digested on the column using Lysyl Endopeptidase (Lys C, Wako), and trypsin (Promega) before elution from the columns and measurement of peptide concentration (Pierce Quantitative fluorometric Peptide Assay).

A total of 9 samples each containing 35 μg peptides from one male and one female mouse of the same genotype (a total of 70 μg peptide) were labelled for TMT according to the manufacturer’s instructions (n = 4 for Ncbe ko, and n = 5 for Ncbe wt, TMT10plex™ Label Reagent Set, Thermo Fisher Scientific). The total amount of protein in Ncbe ko samples was 68.7 ± 2.9% of Ncbe wt (100 ± 3.9%, p = 8.3 × 10^−6^). Equal amounts of peptides from each labelling reaction were pooled, and the sample was desalted on a C18 column (Oasis HLB 1 cc Extraction Cartridges, Waters). The 128C channel was used as control label to normalize across different mass spectrometry runs (denominator). All other channels were compared to the 128C channel to calculate a normalized ratio.

### Mass spectrometry (MS) analysis

The TMT labelled samples were analysed by nano Liquid chromatography (nLC) (easy LC 1000, Thermo Fisher) coupled to a mass spectrometer (Q Exactive, Thermo Scientific) through an EASY-Spray nano-electrospray ion source (Thermo Scientific). A pre-column (Acclaim^®^PepMap 100, 75 µm × 2 cm, C18, 3 µm, 100 Å, Thermo Scientific) and analytical column (EASY-Spray Column, PepMap, 75 µm x 25 cm, C18, 3 µm, 100 Å, Thermo Scientific) were used to trap and separate peptides, respectively. For nLC separation, buffer A was 100% H_2_O/0.1% formic acid and buffer B was 100% ACN/0.1% formic acid. A linear gradient from 5% to 20% buffer B for 24 min, and then from 20% to 35% for 12 min were used for separation of peptides. Precursor scan was performed at a resolution of 70,000, maximum injection time of 100 ms and automatic gain control of 3 × 10^6^. Up to 10 data-dependent tandem mass spectrometry (MS/MS) scans were performed at a resolution of 35,000, maximum injection time of 100 ms and AGC of 1 × 10^5^. HCD normalized collision energy (NCE) was set at 30% with stepped NCE of 20%. Fixed first mass was set at 115. Dynamic exclusion of 30 s as well as rejection of precursor ions with charge state + 1 and above + 8 was employed.

### MS data analysis

Raw files were searched against a mouse protein database (RefSeq database downloaded Nov. 2015 containing 57925 sequences) using both the Sequest and Mascot algorithms (version 2.5.1, Matrix Science) through Proteome Discoverer software (version 2.1, Thermo Scientific). Precursor mass tolerance was set as 10 ppm and fragment mass tolerance was set as 0.02 Da, and a maximum of 2 miss cleavage sites. Carbamidomethylation of cysteine was set as a static modification. N-terminal acetylation, methionine oxidation, TMT labelling of N-terminus and lysine, as well as phosphorylation of serine, threonine and tyrosine were set as variable modifications. False discovery rate (FDR) of 1%, calculated by Percolator, was employed as the cut-off. Peptides identified and quantified in all channels were subjected to Benjamini–Hochberg (BH) FDR estimations, and those that passed the 1% BH-FDR threshold were retained for further analysis. Gene Ontology annotation and analysis was performed with PANTHER (Protein ANalysis THrough Evolutionary Relationships) Classification System version 11 [16, 17].

### Immunoblotting

Dissected mouse choroid plexi were dissolved in ice-cold dissection buffer containing 0.3 M sucrose, 25 mM imidazole, 1 mM EDTA, 8.4 μM leupeptin (Calbiochem), and 0.4 mM Pefabloc (Roche), with pH 7.2, and sonicated using a probe sonicator (BioLogics Inc. 150 V/T, 3 × 5 bursts at 60% power). Protein contents were quantified (Pierce BCA Protein Assay Kit) and samples were adjusted to 1.5% (wt/vol) sodium dodecyl sulfate, 40.0 mM 1,4-dithiothreitol, 6% (vol/vol) glycerol, and 10 mM Tris, pH 6.8 with bromophenol blue. The samples were heated at 65 °C for 15 min and approximately 10 µg of protein per sample was separated by 12.5% polyacrylamide gel electrophoresis and electrotransferred onto PVDF membranes (Ambion). The membranes were blocked with 5% milk in PBS-T (PBS with 0.1% vol/vol Tween), and incubated overnight at 4 °C with primary antibody (Table 1) in PBS containing 1% bovine serum albumin (BSA) and 2 mM NaN_3_. After extensive washing in PBS-T, the blots were incubated with horseradish peroxidase-conjugated anti-rabbit secondary antibody (Dako), washed again in PBS-T and developed with ECL before imaging (ImageQuant LAS4000, GE Healthcare). The CP from one mouse only yields in the range 60–100 µg of protein sample. To increase the number of blots per experimental animal, the membranes were divided for high-, medium-, and low-molecular proteins prior to antibody incubation. For semi-quantitation, band intensities were normalized to the immunoblot signal for proteasome 20 s for the same membrane and lane.

**Table 1:** Primary antibodies used in this study

Target	Antibody number	Host	Source
Ncbe	1139AP	Rabbit	Own laboratory
AQP1	2353AP	Rabbit	Own laboratory
Na,K-ATPase β1	SpET β1	Rabbit	Martín-Vasallo [39]
Na,K-ATPase α1	3B-0/56-0	Mouse	Forbush, 3rd [40]
Na,K,Cl cotransporter 1	N-term. NKCC1	Rabbit	Turner [41]
Anion exchanger 2	9899 C-terminal	Rabbit	Alper [42]
α2-spectrin	LS-C137722	Rabbit	LifeSpan Biosciences
Ankyrin-3	sc-28561(H-215)	Rabbit	Santa Cruz Biotech
P-cadherin	PAB013Mu01	Mouse	Cloud Clone Corp.
α-catenin	LS-B4457	Goat	LifeSpan Biosciences
β-catenin	sc-7199(H-102)	Rabbit	Santa Cruz Biotech
Ezrin	sc-6409 (C-15)	Goat	Santa Cruz Biotech
Moesin	ab50007	Mouse	Abcam
α-adducin	sc-25731 (H-100)	Rabbit	Santa Cruz Biotech
PGC-1α	MBS840561	Rabbit	MyBiosource
TUFM	HPA018991	Mouse	Atlas Antibodies
CTCF	sc-271474	Mouse	Santa Cruz Biotech
Cytochrome C	sc-13156	Mouse	Santa Cruz Biotech
Glycogen phosphorylase	LS-B13107-50	Rabbit	LifeSpan Biosciences
pS373SPAK/pS325OSR1	07-2273	Rabbit	Millipore
pS380SPAK/p332OSR1	pS380-SPAK	Rabbit	Shibuya [43]
IRBIT (AHCYL1)	sc-271581	Mouse	Santa Cruz Biotech
Proteasome 20s	Ab3325	Rabbit	Abcam

### Tissue fixation and immunohistochemistry

Mice were perfusion fixed via the heart with 4% paraformaldehyde in PBS. After fixation, the brain was removed, post-fixed for 2 h, dehydrated in EtOH and xylene, and embedded in paraffin wax, enabling 2 μm sectioning using a rotary microtome (Leica). The sections were de-waxed and stepwise rehydrated, before epitopes were retrieved by boiling the sections in TEG buffer: 10 mM Tris buffer with 0.5 mM EGTA (pH 9), or in 10 mM citrate-buffer (pH 6). The epitopes were quenched with 50 mM NH_4_Cl in PBS, and unspecific binding was blocked by washing with 1% BSA in PBS with 0.2% gelatin and 0.05% saponin. Sections were incubated overnight at 4 °C with primary antibody diluted in 0.1% BSA in PBS with 0.3% Triton X-100. Primary antibodies are listed in Table 1. For fluorescence visualization of the primary antibodies, AlexaFluor 488- or 555-coupled donkey anti-goat, -rabbit, or -mouse secondary antibodies (Invitrogen) were used, and cell nuclei were visualized using Topro-3 counterstaining (Invitrogen). Sections were mounted with a coverslip in Glycergel anti-fade medium (DAKO) and analyzed using a Leica DMIRE2 inverted microscope with a TC5 SPZ confocal unit using a × 63/1.32 NA HCX PI Apo oil objective.

### Image analysis

Protein abundance was investigated by quantifying the immunofluorescence intensities from confocal micrographs. All tissues were carefully handled in parallel from the time of fixation throughout embedding, sectioning, staining, and imaging. To avoid saturation of the photomultiplier, the intensity dynamic range (gain and offset) was adjusted to span the intensities of the most intense sample for each antibody. Images were acquired in the focal plane with the highest signal intensity using fixed settings for magnification, laser power, gain, image depth, offset, and averaging for all images with a given antibody.

The immunofluorescence intensities of the stained tissue was quantified from gray-scale images using Image Pro (Media Cybernetics). For each image, the area of interest was manually defined to avoid counts from non-choroidal tissue or artifacts (Additional file 1: Figure S1). A binary mask of the total area of interest was produced from the fluorescence image. Finally the minimal value for each image pixel was obtained allowing calculation of the total fluorescence count above background within the area of interest (the specific epithelial immunolabeling). For all quantifications, the fluorescence signal was normalized to cell numbers by counting nuclei within the area of interest. All analyzed images were from 4th ventricle CP. In Scatter plots, data are normalized to the mean wild type fluorescence signal. A similar strategy was applied to estimate the average epithelial cell areas as a proxy for cell volume.

### Adenosine triphosphate (ATP) assay, and K^+^ content measurements

Mice were euthanized under isoflurane anaesthesia and CP from all four ventricles of each mouse were isolated in 4 °C Hepes-buffered salt solution (HBS, pH 7.4). Each CP tissue sample was weighed, snap-frozen in liquid nitrogen for cell lysis, added 100 μL Milli-Q water and boiled for 1 min to inhibit ecto-ATPase activity. A fraction of the samples were used for an ATP-determination assay using firefly luciferase. The luminescent signal was recorded on a plate reader (Mithras LB 940, Berthold Technologies). The remainings of the solubilized CP samples were diluted and used for measuring Na^+^ and K^+^ levels by flame photometry (FLM3, Radiometer).

### Co-immunoprecipitation

CPs were lysed in 50 mM Tris–HCl pH 7.4, 150 mM NaCl, 0.25% Na-Deoxycholate, 1% Triton X-100, 1 mM EDTA, 20 mM *N*-ethylmaleimide, containing protease inhibitors Leupeptin and Phefa-block (Boehringer Mannheim) and phosphatase inhibitor cocktail tablets (PhosSTOP, Roche Diagnostics). Following sonication, samples were centrifuged at 10,000×*g* for 10 min at 4 °C. Samples were assayed for protein concentration and immunoprecipitation was performed at 4 °C for 1 h using ∼ 150 µg of lysate and 1 µg of anti-Ncbe or anti-proteasome 20 s antibodies in a total volume of 500 µL. Lysates were subsequently incubated with 20 µL of protein-A-agarose (Santa Cruz Biotechnology) followed by washing three times with lysis buffer and elution in sample buffer and processed for mass spectrometry.

### Proximity ligation assay (PLA)

Immunolabeling was performed as above, but instead of secondary antibody, PLA MINUS and PLUS probes were added, and the labelling with orange detection reagent was performed according to manufacturer’s protocol (Duolink, Sigma). Images were acquired as above with 543 nm laser excitation and emission range 570–650 nm.

### Statistical analysis

A two-tailed t-test was used to compare changes between groups (For MS data analysis: Excel, Microsoft, and for all other data: InStat, GraphPad Software). A level of p < 0.05 was considered adequate to indicate statistical significance. The exact p-values for mass spectrometry analysis are shown in tables or in the linked database.

## Results

### Ncbe ko greatly alters the general protein expression profile in the CP

**Fig. 1:**
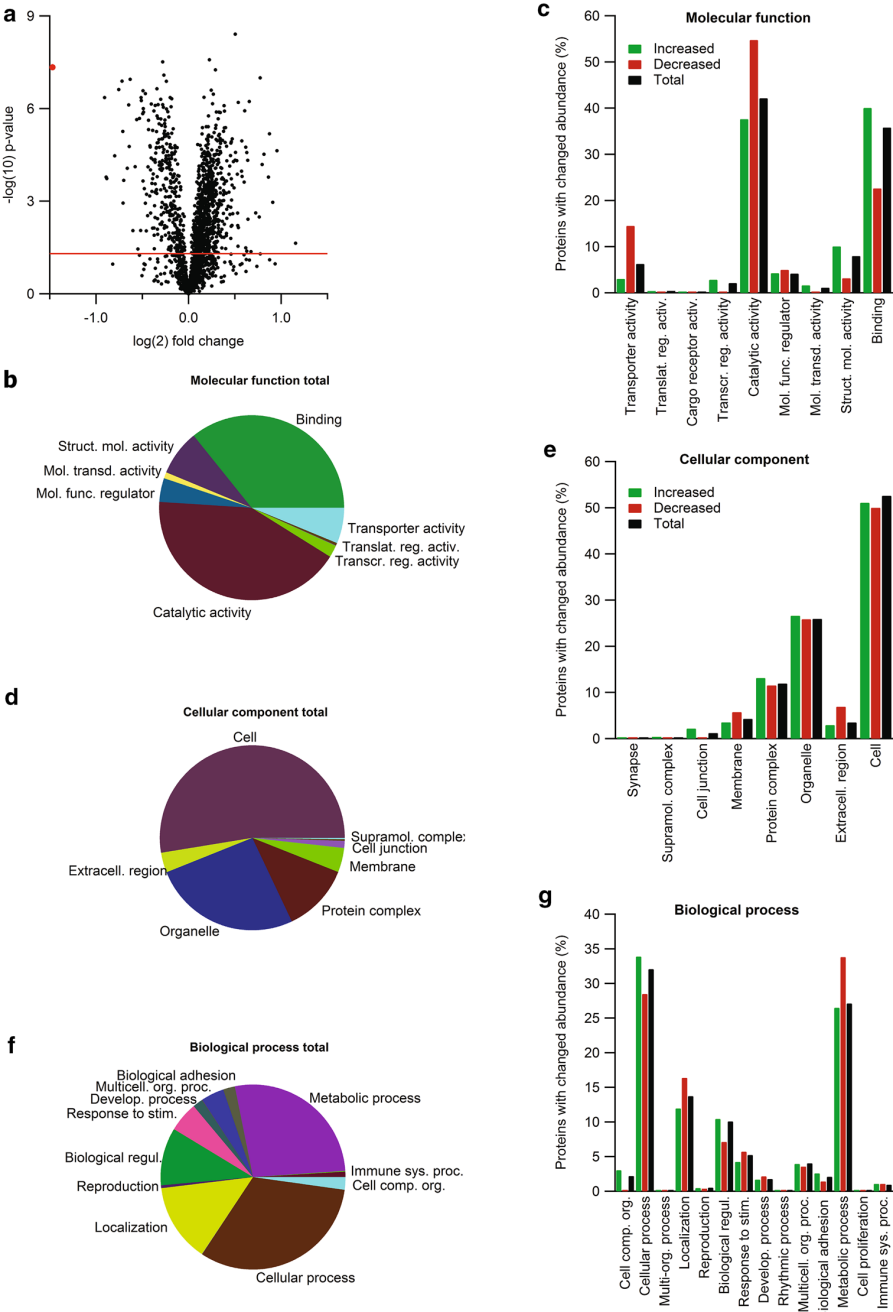
Proteomic and bioinformatic profile of the CP from Ncbe wt and Ncbe ko mice. **a** Volcano plot of the peptide quantification in Ncbe wt and Ncbe ko CP, where the primary axis shows the log_2_ (mean peptide abundance ratio), while the secondary axis designates the −log_10_(p value). The horizontal red line represents the Benjamini–Hochberg false discovery rate threshold (p = 0.05). The red dot marks the data point for Ncbe peptides encoded by pre-STOP codons. Pie and bar graphs visualizing the distribution of proteins detected in both groups classified by gene ontology (GO) terms for molecular functions (**b**, **c**), cellular components (**d**, **e**), and biological processes (**f**, **g**). The bar graphs illustrate the percent wise distribution of proteins among GO-terms topics for all detected proteins (black bars), and proteins that are either increased (green bars) or decreased (red bars) in Ncbe ko CP compared to Ncbe wt

Mass spectrometry combined with a TMT labeling strategy identified 1865 proteins in all 9 samples. Of these, 601 were significantly changed in abundance in Ncbe ko CP (390 proteins increased, 211 proteins decreased). 340 proteins were increased in abundance by more than 10%, of which 86 were increased more than 25%. Conversely, 183 proteins decreased in abundance by more than 10%, of which 34 decreased by more than 25%. Thus, in general, more proteins were increased in abundance than decreased in Ncbe ko CP, despite an appropriate centering of the data around zero-fold change in the volcano plot (Fig. 1a).

Table 2 lists the 20 proteins with lowest relative abundance in Ncbe ko cells compared to Ncbe wt cells isolated from the CP. The protein most affected by deletion of *slc4a10* is, as expected, Ncbe itself. Several N-terminal peptides of Ncbe can still be detected in the Ncbe ko, as the stop codon in the gene modified mouse was introduced after the N-terminus coding region. A total of 8 plasma membrane transport proteins including 4 Na^+^,K^+^-ATPase subunits (α 1, α4, β1, and phospholemman), and 5 enzymes—4 of which are involved in ATP synthesis or transport—are also decreased in abundance. In general, these results support the idea of a decreased capacity for secretion and ATP production, respectively, in Ncbe ko cells. Five proteins normally originating from the blood (the hemoglobins and anti-trypsins), were also detected previously in a proteomic study of FACS isolated single CPECs [18], along with the neuronal membrane glycoprotein M6-a.

**Table 2:** Proteins with lowest relative abundance in CP from Ncbe ko compared to Ncbe wt mice

Accession	Description	p-value	Ratio
334688852	Sodium-driven chloride bicarbonate exchanger 1	4.57E−08	0.36
6753138	Sodium/potassium-transporting ATPase subunit β-1	4.40E−07	0.53
145301549	Hemoglobin α, adult chain 2	1.66E−04	0.54
31982300	Hemoglobin, β adult t chain	1.84E−04	0.54
254588010	Aminopeptidase Q	3.41E−05	0.57
21450277	Sodium/potassium-transporting ATPase subunit α-1	2.42E−07	0.59
755519908	Phospholemman X1	1.30E−07	0.61
23957686	Neuronal membrane glycoprotein M6-a1	5.49E−06	0.61
209863008	V-type proton ATPase 116 kDa subunit a3	1.16E−03	0.61
498752597	Hemoglobin subunit β-1	2.12E−04	0.62
357588427	α-1-antitrypsin 1-1 2	8.64E−05	0.63
91598783	Solute carrier family 28 member 3	7.64E−07	0.64
6681095	Cytochrome c	1.70E−05	0.64
22094075	ADP/ATP translocase 2	1.13E−07	0.65
6680710	Aquaporin-1	7.69E−05	0.67
6678085	Alpha-1-antitrypsin 1-4	2.29E−06	0.68
16716343	Cytochrome c oxidase subunit 6C	2.09E−06	0.69
33563266	Cytochrome c oxidase subunit NDUFA4	1.46E−06	0.71
20330802	Serotransferrin precursor	5.53E−07	0.70
226958351	Sodium/potassium-transporting ATPase subunit α-4	4.40E−07	0.70

The accession numbers are shown along with the protein names (description and common abbreviation), the t-test results, and the abundance ratio Ncbe ko/Ncbe wt

Table 3 lists the 20 proteins with the highest relative abundance in Ncbe ko CP compared to Ncbe wt CP. Four of these proteins are involved in regulation or protein breakdown (OSR1, IRBIT, Ceacam-2 and the E2 ligase), and one is a chromosomal protein (HMG-17). Additionally, one protein is involved in glycogen metabolism and one in glucuronidation of water insoluble substances. The two proteins in the list normally arising from neurons or glia (Purkinje cell protein 4 and glial fibrillary acidic protein 2, respectively) were both detected previously in FACS isolated single CPECs [18]. These proteins are therefore considered expressed by CPECs or taken up by the epithelial cells or Kolmer cells (choroid plexus macrophages), which would be the only probable contaminating cell in the referred study.

**Table 3:** Proteins with highest relative abundance in CP from Ncbe ko compared to Ncbe wt mice

Accession	Description	p-value	Ratio
164565419	Carcinoembryonic antigen CAM 2 (Ceacam-2)	2.32E−05	1.94
6755592	γ-synuclein	1.08E−03	1.88
8393534	Non-histone chromosomal protein HMG-17	6.57E−06	1.83
6679227	Purkinje cell protein 4	1.66E−04	1.82
31542956	Ubiquitin-conjugating enzyme E2 K 1	3.02E−05	1.76
145699099	UDP-glucuronosyltransferase 1-1	6.49E−05	1.73
27734986	Putative adenosylhomocysteinase 2 (IRBIT)	1.01E−07	1.71
84000448	Glial fibrillary acidic protein 2	3.26E−03	1.70
568963419	Serine/threonine-protein kinase OSR1	2.40E−03	1.65
569018439	Prostaglandin F2 receptor negative regulator	5.92E−07	1.58
24418919	Glycogen phosphorylase, brain form	4.75E−05	1.56
124486606	Predicted gene 12657	2.66E−05	1.55
257900524	MAGUK p55 subfamily member 6	1.78E−05	1.53
84042521	Barrier-to-autointegration factor	1.19E−06	1.52
569010320	Disks large homolog 3	2.40E−04	1.52
10048452	Monocarboxylate transporter 3	1.59E−04	1.49
161353454	CD151 antigen	1.11E−05	1.49
6680359	Interferon gamma inducible protein 47	1.07E−04	1.49
166235890	Pro-cathepsin H isoform 1 preproprotein	6.30E−07	1.45
568935961	Septin-11 isoform X1	2.42E−03	1.44

The accession numbers are shown along with the protein names (description and common abbreviation), the t-test results, and the abundance ratio Ncbe ko/Ncbe wt

All detected CP proteins were classified using Gene Ontology (GO) annotation. Figure 1b–g show the distribution of the identified proteins among the GO-terms within the groups: molecular function, cellular component, and biological process, respectively. For molecular function, the proteins were predominantly enzymes, molecule binding proteins, structure molecules, and transporters (Fig. 1b). Figure 1c highlights that more proteins involved in binding and structure molecules are increased in Ncbe ko compared to Ncbe wt. For catalytic activity and transporter activity, the opposite is observed; more proteins are decreased than increased in abundance in Ncbe ko compared to Ncbe wt. Cell structures, organelles, macromolecular complexes and membrane proteins dominated the group “cellular processes” (Fig. 1d). Figure 1e illustrates that there are similar numbers of increasing and decreasing proteins in each group except for the “membrane” and “extracellular region” groups. Here, more proteins decrease in abundance rather than increase in Ncbe ko CP compared to Ncbe wt CP. For biological processes, most proteins belonged to metabolic processes, cell processes, and cell architecture (Fig. 1f). As shown in Fig. 1g, more proteins annotated to metabolic processes and biological regulation decrease than increase in abundance in Ncbe ko. The opposite is observed for cellular processes and cellular organization. Taken together, the data indicate that Ncbe ko cells have less catalytical or metabolical capacity, are less secretory active than Ncbe wt cells, and that structural and morphological changes occur in Ncbe ko CPECs.

### Changes in transporter abundance indicate a lower secretory capacity in the Ncbe ko CP

**Fig. 2:**
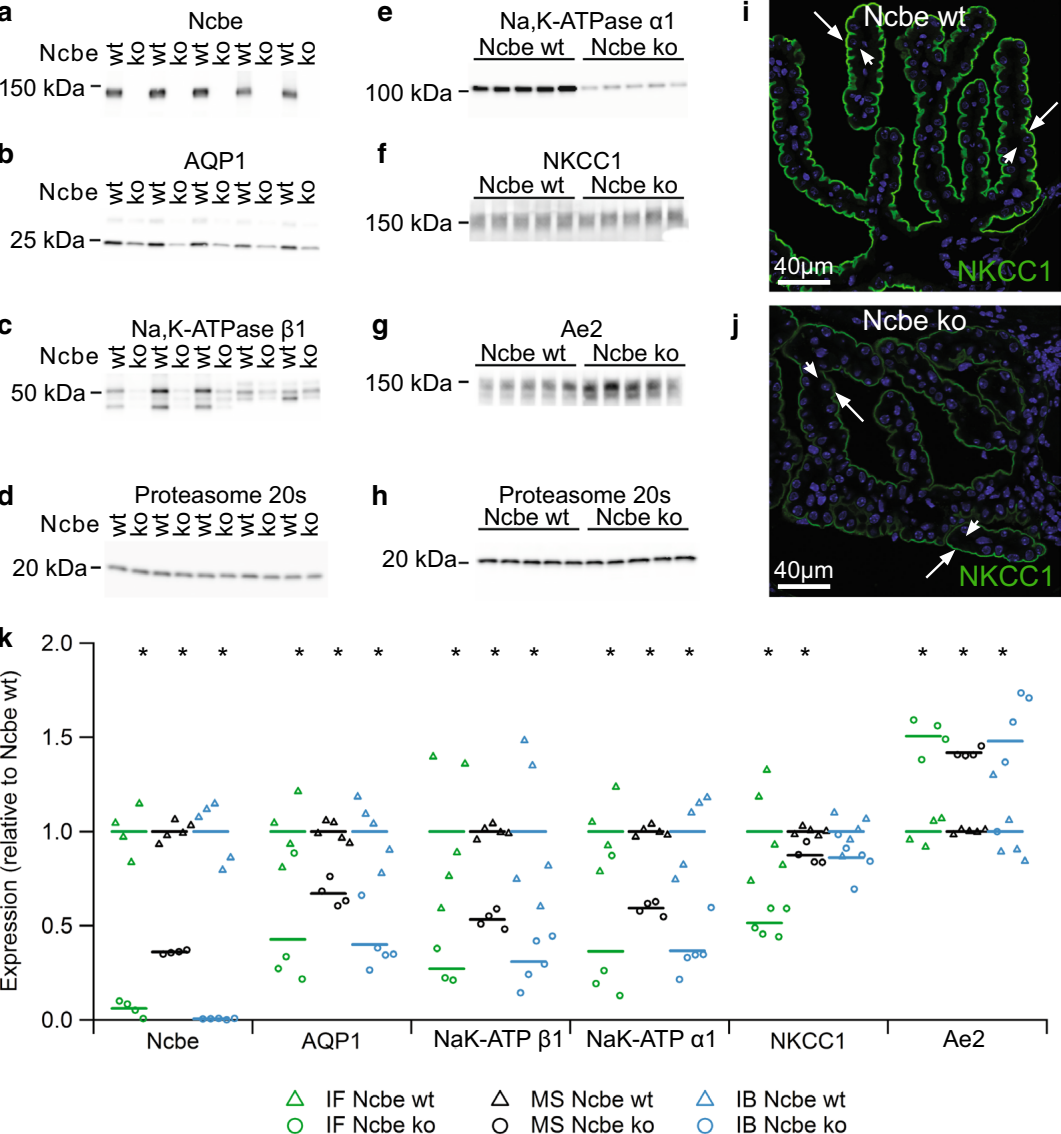
Analysis of plasma membrane transporter expression. Immunoblot analysis of the protein abundance in the CP from Ncbe wt and Ncbe ko mice for **a** Ncbe, **b** AQP1, **c** Na,K-ATPase β1 subunit, **d** Proteasome 20 s, **e** Na,K-ATPase α1 subunit, **f** NKCC1, **g** Ae2, and **h** Proteasome 20 s. Immunofluorescence histochemistry was applied to compare protein expression of NKCC1 (green) in the IVth ventricle CP from **i** Ncbe wt and **j** Ncbe ko mice. Arrows indicate the luminal plasma membrane, while arrowheads indicate the basolateral membrane labyrinth. Nuclei are stained blue. **k** Scatter plot comparing the relative changes in transporter protein abundance obtained by immunofluorescence microscopy (IF), proteomic mass spectrometry analysis (MS), and immunoblotting (IB) (*p < 0.05, n = 5). Mean values are normalized to control (Ncbe wt) and indicated by horizontal bars. Triangles indicate data points from Ncbe wt CP, whereas circles represent data from Ncbe ko CP. Mean data for the IF semi-quantitation, except for NKCC1 are from previous publications [13–15]

Plasma membrane transporters were a major class of altered proteins in CPECs from Ncbe ko mice). Many of the proteins with decreased abundance in Ncbe ko mice are closely associated to the secretion of cerebrospinal fluid by the CP. While the abundance of the classical epithelial Na^+^,K^+^-ATPase subunits α1 and β1 were reduced to approximately 60% in the Ncbe ko, the alternative α2, α3 and β2 subunits were increased by 10–20% in Ncbe ko than Ncbe wt CP. Figure 2a–h shows immunoblot validation of the TMT 10-plex data for 5 transporters with decreasing abundance and 1 transporter with increased abundance along with proteasome 20 s loading controls (Ncbe p < 0.0001; AQP1, p = 0.0003; Na^+^,K^+^-ATPase β1 p = 0.0055; Na^+^,K^+^-ATPase α1 p = 0.0014; NKCC1 p = 0.059; Anion exchanger 2, Ae2, p = 0.0171; n = 5). Apart from NKCC1, immunofluorescence histochemistry was previously used to determine changes in the abundance of these transporters in the same mouse model [13–15]. Figure 2i, j show representative images used for semi-quantification illustrating reduced NKCC1 abundance in Ncbe ko vs. Ncbe wt CP. Data obtained by immunofluorescence microscopy, mass spectrometry, and immunoblotting are compared in Fig. 2k (for NKCC1 immunofluorescence: p = 0.0030, n = 5). There is a general agreement among the techniques except for NKCC1, where immunofluorescence microscopy seems to overestimate the change in protein abundance. The discrepancy might arise from poor recognition of a cytoplasmic pool of the protein that would be detected by both mass spectrometry and immunoblotting, such as phosphorylated forms of the protein. Despite discrepancies for a few proteins, there seems to be an acceptable correlation among the changes in abundance using the three techniques (Additional file 2: Figure S2).

### Enzymes involved in energy metabolism are prominently affected by Ncbe ko

**Fig. 3:**
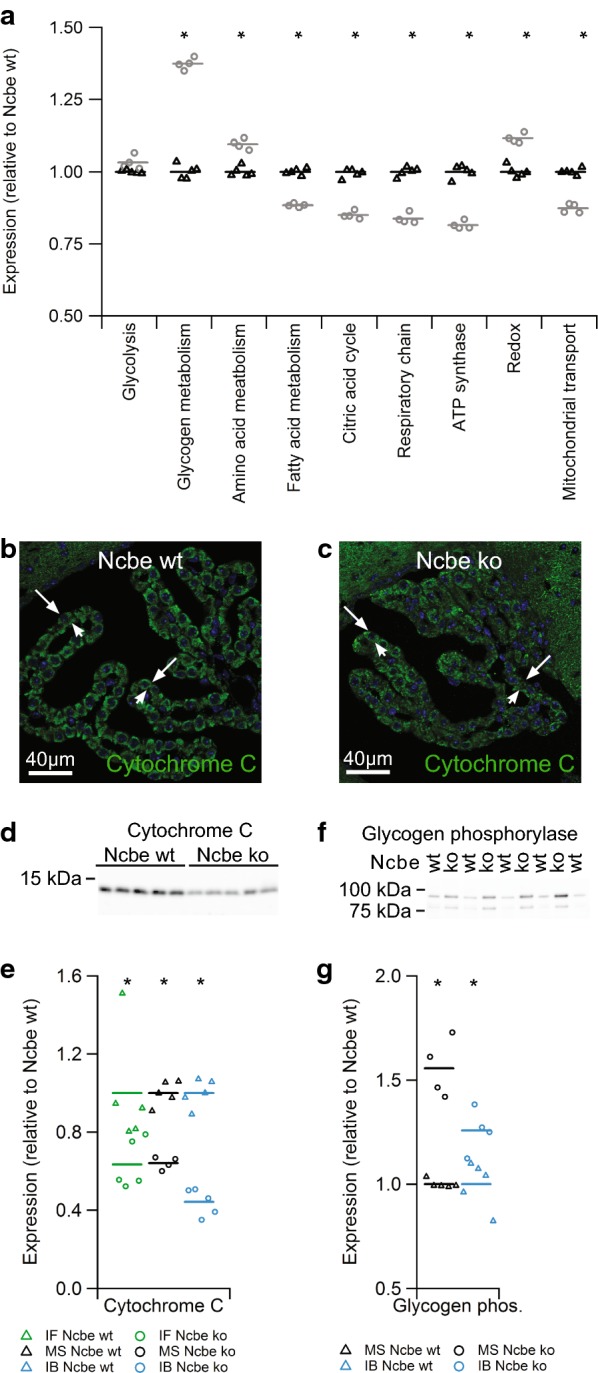
Protein abundance of proteins in selected metabolic pathways. **a** Scatter plot showing the relative changes in abundance between Ncbe wt and Ncbe ko CP among proteins involved in the glycolysis, glycogen, amino acid, and fatty acid metabolism, the tricarboxylic acid (TCA) cycle, respiratory chain, subunits of the ATP synthase, redox enzymes and mitochondrial transport proteins (*p > 0.05, n = 5). Mean values are normalized to control (Ncbe wt) and indicated by horizontal bars. Triangles indicate data point from Ncbe wt CP, whereas circles represent data from Ncbe ko CP. **b**, **c** Representative immunofluorescence micrographs comparing the protein expression of cytochrome C (green) in Ncbe wt and Ncbe ko 4th ventricle CP, respectively. Nuclei are stained blue. **d** Immunoblot analysis of cytochrome C protein abundance in the CP from Ncbe wt and Ncbe ko mice. **e** Scatter plot comparing relative changes in cytochrome C protein abundance obtained by immunofluorescence microscopy (IF), proteomic mass spectrometry analysis (MS), and immunoblotting (IB) (*p < 0.05, n = 5). Mean values are normalized to control (Ncbe wt) and indicated by horizontal bars. Triangles indicate data points from Ncbe wt CP, whereas circles represent data from Ncbe ko CP. **f** Immunoblot analysis of glycogen phosphorylase (brain type) protein abundance in the CP from Ncbe wt and Ncbe ko mice. **g** Scatter plot comparing relative changes in glycogen phosphorylase abundance obtained by proteomic mass spectrometry analysis (MS), and immunoblotting (IB) (*p < 0.05, n = 5). Mean values are normalized to control (Ncbe wt) and indicated by horizontal bars. Triangles indicate data points from Ncbe wt CP, whereas circles represent data from Ncbe ko CP

Numerous proteins involved in cellular metabolism have significantly altered abundance in Ncbe ko vs. Ncbe wt CP. Figure 3a shows the relative abundances of groups of proteins relating to the indicated metabolic pathways. Phosphofructokinase is the rate-limiting step in the glycolysis and all three isoforms detected in CPs have higher abundance in Ncbe ko than Ncbe wt, although the platelet form did not pass the 1% false discovery rate cut-off (Additional file 3: Figure S3). Immunoblotting determined that both the muscle and the neuronal type of the enzyme have higher abundance in Ncbe ko than Ncbe wt (not shown). In contrast to the proposed difference in capacity for respiratory ATP synthesis, thus, the capacity for glycolytic ATP synthesis seems conserved in the Ncbe ko CP.

A large number of mitochondrial enzymes are decreased in abundance in the CP of Ncbe ko mice compared to Ncbe wt. Additional file 4: Figure S4 and Additional file 5: Figure S5 compares the protein levels of detected enzymes related to the citric acid cycle and oxidative phosphorylation in Ncbe ko and Ncbe wt mice, respectively. The pyruvate dehydrogenases and with very few exceptions the entire range of enzymes in the citric acid cycle are uniformly decreased in abundance in the Ncbe ko (Additional file 4: Figure S4). For comparison, the cytoplasmic malate dehydrogenase was increased rather that decreased as opposed to the mitochondrial form. Most detected polypeptides belonging to the complex I and all of the proteins of complexes II, III, and IV of the respiratory chain were also uniformly decreased in abundance in Ncbe ko CP (Additional file 5: Figure S5A, B). On average, the protein with the greatest difference in abundance in Ncbe ko compared to Ncbe wt is cytochrome C. The lower abundance of this cytochrome in Ncbe ko CPECs was confirmed by immunofluorescence microscopy (Fig. 3b, c) and immunoblot analysis (Fig. 3d). Comparative analysis among the three different techniques in shown in Fig. 3e, where the agreement among the techniques is high (p = 0.0001 for immunoblotting and p = 0.0329 for immunofluorescence microscopy, n = 5).

The abundances of specific enzymes involved in glycogen metabolism are shown in Additional file 3: Figure S3B. Glycogen phosphorylase is a member of the metabolic enzymes with the highest abundance in Ncbe ko compared to Ncbe wt (Fig. 3f, p = 0.009876; n = 5). Three isoforms of the enzyme were identified in the CP, with the brain type glycogen phosphorylase in Ncbe ko mice increased as assessed by both mass spectrometry and immunoblotting (Fig. 3g). The abundance of enzymes involved in glycogen synthesis, such as glycogen synthase, are also elevated in Ncbe ko mice (Additional file 3: Figure S3B). Thus, the capacities for both synthesis and breakdown of glycogen is likely increased in the Ncbe ko mouse model. Additional file 3: Figure S3C shows that enzymes of fatty acid metabolism are generally decreased in abundance in the Ncbe ko CP compared to Ncbe wt, while the four detected enzymes involved in amino acid metabolism are increased in abundance in Ncbe ko (Additional file 3: Figure S3D).

Numerous subunits of the ATP synthase complex were detected by mass spectrometry (Additional file 6: Figure S6A). Except for one, all of these proteins are lower in abundance in CP isolated from Ncbe ko mice relative to Ncbe wt. Thus, the cellular capacity for mitochondrial ATP synthesis seems decreased in CPECs from Ncbe ko compared to Ncbe wt mice. The more variable decrease in enzymes of the citric acid cycle compared to the respiratory chain and ATP synthase may reflect their participation in a variety of other metabolic pathways other than oxidative phosphorylation.

**Fig. 4:**
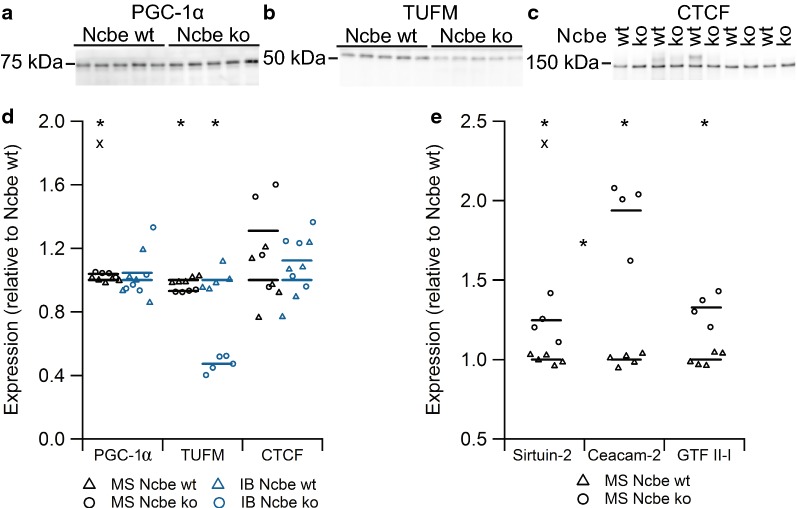
Analysis of the relative expression of proteins involved in the regulation of energy metabolic pathways. Immunoblot analysis of the protein abundance in the CP from Ncbe wt and Ncbe ko mice for **a** PGC-1α, **b** TUFM, and **c** CTCF. **d** Scatter plot comparing relative changes in protein abundance obtained by proteomic mass spectrometry analysis (MS), and immunoblotting (IB) (*p < 0.05, X: Failed FDR of 1%, n = 5). Mean values are normalized to control (Ncbe wt) and indicated by horizontal bars. Triangles indicate data point from Ncbe wt CP, whereas circles represent data from Ncbe ko CP. **e** Scatter plot showing the comparative mass spectrometry analysis of additional regulatory proteins Sirtuin-2, Ceacam-2 and general transcription factor II-I between Ncbe wt and Ncbe ko CP (*p < 0.05, X: Failed FDR of 1%, n = 5). Mean values are normalized to control (Ncbe wt) and indicated by horizontal bars. Triangles indicate data points from Ncbe wt CP, whereas circles represent data from Ncbe ko CP

Many molecular pathways are involved in transcriptional regulation of cellular metabolic activities. We assessed the expression levels of three of such pathways by immunoblotting (Fig. 4a–c) and compared the results to those obtained by mass spectrometry data (Fig. 4d). Peroxisome proliferator-activated receptor γ coactivator 1α (PGC-1α) is a transcriptional coactivator in mitochondrial biogenesis and oxidative metabolism that is highly sensitive to the energy status of the cell and under the control of the AMP-activated protein kinase (AMPK) [19]. PGC-1α abundance was unchanged with both mass spectrometry and immunoblotting (Fig. 4a, d, for immunoblotting p = 0.6472, n = 5), indicating that CPECs from Ncbe ko mice. Indeed, the negative regulator of PGC-1α, sirtuin-2 display a tendency towards a higher abundance in Ncbe ko cells compared to Ncbe wt by mass spectrometry (Fig. 4e). The carcino-embryonic antigen-related cell adhesion molecule 2 (Ceacam-2) has been linked to a decrease in energy use at the organism level [20, 21], and is almost doubled in abundance in Ncbe ko CP compared to Ncbe wt (Fig. 4e).

Mitochondrial Tu translation elongation factor (TUFM) is a key factor in translation of mitochondrial DNA, thereby playing an important role in the control of mitochondrial function [22]. TUFM abundance was slightly decreased in Ncbe ko CP compared to Ncbe wt as assessed by mass spectrometry, but was more than halved in abundance as assessed by immunoblotting (Fig. 4b, d, for immunoblotting p = 0.0001, n = 5). This may indicate a lower degree of mitochondrial synthesis in Ncbe ko than in Ncbe wt CP.

The cellular insulator protein, CCCTC-binding factor (CTCF) is a key regulator of nuclear chromatin structure and a transcriptional repressor [23]. It interacts with the general transcription factor II-I (GTF II-I), which serves various roles in transcription and signal transduction, such as directing CTCF to the promoter proximal regulatory regions of genes involved in metabolism. Although CTCF abundance was not statistical significantly changed by immunoblotting (Fig. 4c, p = 0.532, n = 5), both CTCF and GTF II-I levels are higher in Ncbe ko CP than in Ncbe wt by mass spectrometry (Fig. 4d, e). Thus, further studies are needed to discern whether the observed decrease in energy metabolic enzymes are governed by the CTCF/GTF II-I system.

### The CP ATP level is elevated in Ncbe ko mice

**Fig. 5:**
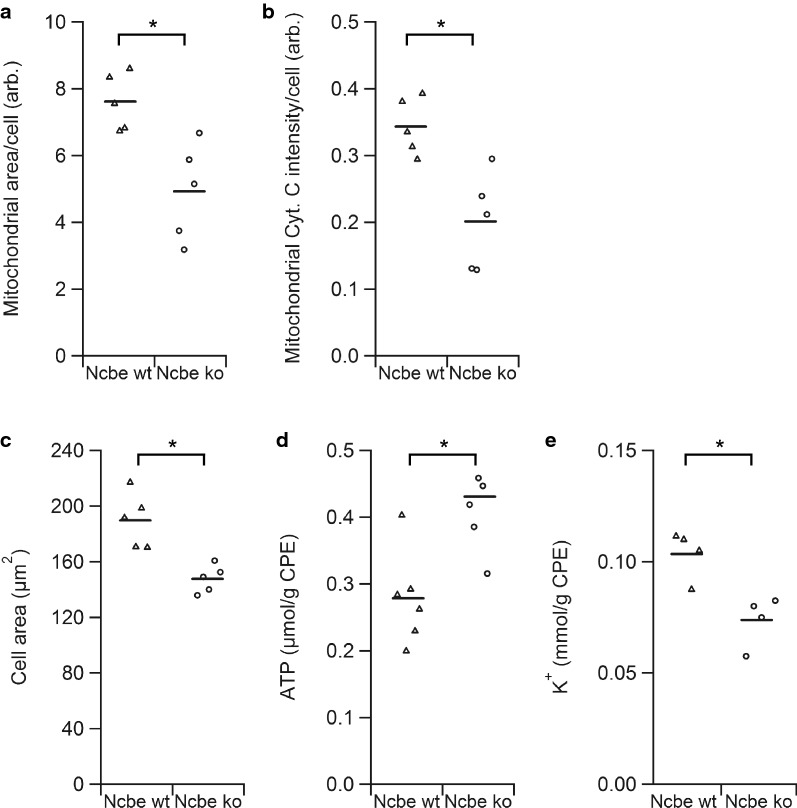
Evaluation of mitochondrial abundance, cell size, ATP, and K^+^ levels. **a** Scatter plot comparing the average mitochondrial area determined by the cytochrome C positive area within cells from Ncbe wt and Ncbe ko CP (*p < 0.05, n = 5, representing 364 Ncbe wt cells and 482 Ncbe ko cells). Mean values indicated by horizontal bars. Triangles indicate data points from Ncbe wt CP, whereas circles represent data from Ncbe ko CP. **b** The mean cytochrome C immunostaining intensity per cell from the Ncbe wt and Ncbe ko CP epithelium, respectively (*p < 0.05, n = 5, representing 364 Ncbe wt cells and 482 Ncbe ko cells). Mean values indicated by horizontal bars. Triangles indicate data points from Ncbe wt CP, whereas circles represent data from Ncbe ko CP. **c** Estimation of the average cell size in Ncbe wt and Ncbe ko CP epithelium from micrographs of background fluorescence (*p < 0.05, n = 5, representing 1214 Ncbe wt cells and 1222 Ncbe ko cells). Mean values indicated by horizontal bars. Triangles indicate data points from Ncbe wt CP, whereas circles represent data from Ncbe ko CP. **d** Scatter plot showing the mean ATP levels in the CP from Ncbe wt and Ncbe ko mice by chemiluminescence (*p < 0.05, n = 6). Mean values indicated by horizontal bars. Triangles indicate data points from Ncbe wt CP, whereas circles represent data from Ncbe ko CP. **e** Estimation of the mean cellular K^+^ content in the CP from Ncbe wt and Ncbe ko mice by flame photometry (*p < 0.05, n = 4). Mean values indicated by horizontal bars. Triangles indicate data points from Ncbe wt CP, whereas circles represent data from Ncbe ko CP

The simplest explanation for the general decrease in mitochondrial enzymes would be a decline in number or—more precisely—the cellular volume of mitochondria in CPECs in Ncbe ko mice compared to Ncbe wt. The predominantly decreased abundance of mitochondrial translocating proteins (Additional file 6: Figure S6B) and the specific decrease in mitochondrial (or type B) vs. cytoplasmic (or type A) cytochrome b5 (Additional file 6: Figure S6C) in CPECs from Ncbe ko mice provide support for this notion. Figure 5a shows the estimated mitochondrial area per cell as assessed by the cytochrome C immunofluorescence signal. The analysis revealed a decrease in cellular mitochondrial area to approximately 65% (p = 0.0073, n = 5, with a total of 482 Ncbe wt cells and 364 Ncbe ko cells assessed) in Ncbe ko CPECs compared to Ncbe wt CPECs. The cellular cytochrome C intensity in the Ncbe ko CPECs cells amounted to approximately 59% of the Ncbe wt values in the same analysis (Fig. 5b, p = 0.0051, n = 5). Thus, both the mitochondrial volume and cytochrome C intensity seems decreased in CPECs from Ncbe ko compared to Ncbe wt. The average area of the epithelial cells from Ncbe ko mice amounted to approximately 78% of the area of CPECs from Ncbe wt mice as assessed from the background fluorescence micrographs of the CP (Fig. 5c, p = 0.0028, n = 5, with a total of 1214 Ncbe wt cells and 1222 Ncbe ko cells assessed). This is equivalent to a decrease to 69% in estimated cell volume from 2611 to 1794 µm^3^. Taking the smaller cell size in Ncbe ko mice into account, the mitochondrial area in the CPECs is reduced to 83% of the Ncbe wt cells, which does not reach statistical significance (p = 0.1982, n = 5).

The cellular ATP levels in Ncbe wt and Ncbe ko CP were compared in order to assess the consequences of the potential decreased cellular capacity for ATP synthesis. Figure 5d shows that the Ncbe ko CP has higher ATP levels compared to the Ncbe wt tissue (p = 0.0060, n = 6). Thus, the Na^+^,K^+^-ATPase expression does not seem to be reduced by low intracellular ATP in the Ncbe ko CP. Low d Na^+^,K^+^-ATPase expression most likely leads to reduced transport activity resulting in a low intracellular K^+^ content. Indeed, the tissue K^+^ content (normally, 97% of K^+^ is intracellular) was significantly reduced in Ncbe ko compared to Ncbe wt (Fig. 5e, p = 0.0092, n = 4), suggesting that less K^+^ is pumped into the cells despite sufficient ATP levels. Thus, the CPECs from Ncbe ko mice appear to maintain a smaller cell volume based on reduced ion transport, but do not lack ATP to drive these processes.

### Altered regulators of ion transporter function in CP from Ncbe ko mice

The “IP3R Binding protein released with Inositol 1,4,5-Trisphosphate”/”Protein Phosphatase-1” (IRBIT/PP1) pathway and the With-No lysine (K)”/”SPS1-related Proline/Alanine-rich Kinase”/“Oxidative Stress–Responsive kinase 1” (WNK/SPAK/OSR1) pathway are important modulators of the cellular ion transporter regulation that likely influence each other at a cellular level [24, 25].

**Fig. 6:**
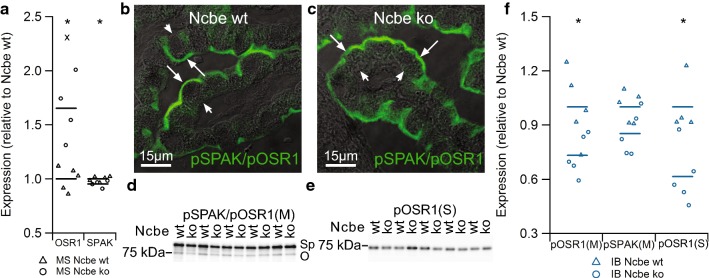
Comparison of the expression of ion transport regulators OSR1 and SPAK in Ncbe wt and Ncbe ko mouse CP. **a** Scatter plot illustrating the difference in mean CP OSR1 and SPAK expression between Ncbe wt and Ncbe ko mice, as assessed by comparative mass spectrometry (*p < 0.05, X: Failed FDR of 1%, n = 5). Mean values are normalized to control (Ncbe wt) and indicated by horizontal bars. Triangles indicate data points from Ncbe wt CP, whereas circles represent data from Ncbe ko CP. **b** Immunofluorescence micrograph showing the cellular distribution of pSPAK/pOSR1 (green) at high magnification of the CP from an Ncbe wt mouse. **c** A similar micrograph of pSPAK/pOSR1 staining in the CP from an Ncbe ko mouse. The fluorescence images are overlaid onto the corresponding differential interference contrast (DIC) images. Arrows indicate the luminal plasma membrane, while arrowheads indicate the basolateral membrane labyrinth. **d**, **e** Immunoblot analysis of the pSPAK/pOSR1 abundance in the CP from Ncbe wt and Ncbe ko mice with two antibodies (“M” and “S”, respectively). Sp indicates the expected migration of pSPAK, whereas O indicates the expected pOSR1 size. **f** Scatter plot comparing relative changes in pOSR1 and pSPAK abundances obtained by immunoblotting (IB) with the two antibodies (*p < 0.05, n = 5). Mean values are normalized to control (Ncbe wt) and indicated by horizontal bars. Triangles indicate data points from Ncbe wt CP, whereas circles represent data from Ncbe ko CP

OSR1 was also in the top-20 percent of proteins with higher abundance in Ncbe ko vs. Ncbe wt CP by mass spectrometry (Table 3, Fig. 6a), while SPAK in Ncbe ko was not significantly changed compared to Ncbe wt CP. However, SPAK was identified in Ncbe co-IP experiments (Additional file 7: Table S1). Antibodies against the phosphorylated OSR1/SPAK (pOSR1 and pSPAK) localized the protein to the luminal membrane domain of CPECs, suggesting a primary function of the active kinase in regulating luminal membrane transporters (Fig. 6b). The staining pattern was conserved in Ncbe wt CP (Fig. 6c). Immunoblot analysis using the common pOSR1/pSPAK antibody indicates that the abundance of pOSR1 (75 kDa band, pSPAK/pOSR1 (M)-antibody) is lower in Ncbe ko than Ncbe wt CP (Fig. 6d, p = 0.02947, n = 5). The decrease in abundance of the higher band representing pSPAK in Ncbe ko CP did not reach statistical significance (p = 0.05899, n = 5), similar to the 65% decrease in immunofluorescence microscopy signal with the same antibody in Ncbe ko tissue (not shown, p = 0.2092). Immunoblotting with a separate antibody [pOSR1 (S)-antibody] that only seem to recognize pOSR1 revealed a similar decrease in pOSR1 abundance in the Ncbe ko tissue (Fig. 6e, p = 0.008258, n = 5). Figure 6f illustrates the lower abundance of pOSR1/pSPAK with the same antibody, pSPAK/pOSR1(M), and pOSR1 with the separate antibody, OSR1(S), in the CP from Ncbe ko mice compared to Ncbe wt mice. Similar results were obtained with three sets of samples, each with n = 5 for each genotype.

**Fig. 7:**
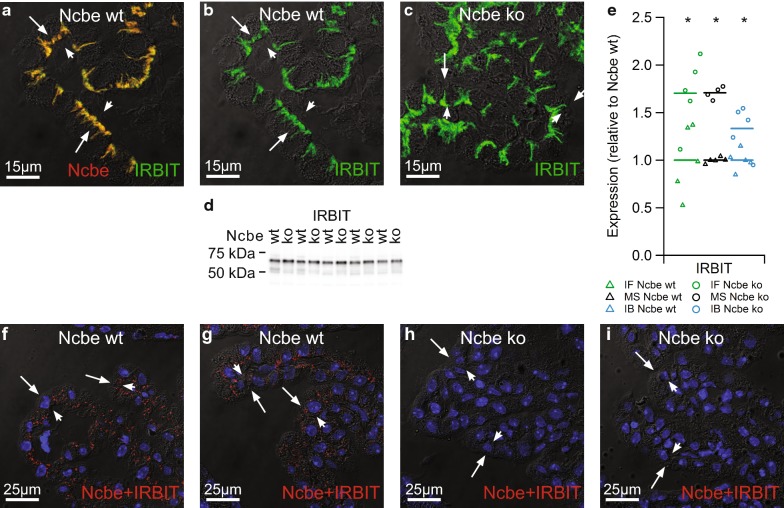
Comparison of the expression of ion transport regulator IRBIT in Ncbe wt and Ncbe ko mouse CP. **a** Double immunofluorescence micrograph stained for Ncbe (red) and IRBIT (green) at high magnification of the CP from a Ncbe wt mouse. **b** The same micrograph showing only the anti-IRBIT immunoreactivity. **c** A similar micrograph of IRBIT staining in the CP from an Ncbe ko mouse. The fluorescence images are overlaid onto the corresponding DIC images. Arrows indicate the luminal plasma membrane, while arrowheads indicate the basolateral membrane labyrinth. **d** Immunoblot analysis of the IRBIT abundance in the CP from Ncbe wt and Ncbe ko mice. **e** Scatter plot comparing relative changes in IRBIT abundance obtained by immunofluorescence microscopy (IF), proteomic mass spectrometry analysis (MS), and immunoblotting (IB)(*p < 0.05, n = 5). Mean values are normalized to control (Ncbe wt) and indicated by horizontal bars. Triangles indicate data points from Ncbe wt CP, whereas circles represent data from Ncbe ko CP. **f**, **g** Representative images resulting from PLA assays using anti-Ncbe and anti-IRBIT antibodies on Ncbe wt CP. **h**, **i** Similar representative images using the same antibodies on Ncbe ko CP (run in parallel). Positive reaction products are shown in red. Nuclei are shown in blue and the fluorescence signals are overlaid onto the corresponding DIC image

IRBIT was immunolocalized to the basolateral membrane domain of the CPECs, where it co-localized with Ncbe (Fig. 7a). IRBIT was also identified using mass spectrometry on Ncbe co-immunoprecipitations (co-IP) from CP samples (Additional file 7: Table S1), suggesting a role of IRBIT in regulating Ncbe. The subcellular distribution of IRBIT was similar in Ncbe wt and Ncbe ko CPECs (Fig. 7b, c). The IRBIT immunofluorescence signal and thereby relative abundance was higher in Ncbe ko CP, which was also observed by immunoblotting (Fig. 7d). Figure 7e compares the IRBIT abundance in Ncbe ko and Ncbe wt CP by semi-quantitative immunofluorescence, mass spectrometry, and immunoblotting. All three techniques revealed a robustly elevated IRBIT abundance in the Ncbe ko CP (p = 0.01725 for immunofluorescence microscopy and p = 0.00865 for immunoblotting, n = 5). PLA assays were executed in order to confirm the close proximity of Ncbe and IRBIT first suggested by the double immunolabeling above. Indeed, positive PLA reaction occurred only in CPECs from Ncbe wt mice, whereas Ncbe ko CPECs were negative (Fig. 7f–i, n = 5). The positive reaction products in Ncbe wt CPECs were mainly, but not exclusively observed in the basolateral membrane domain.

## Discussion

In transporting epithelia, the removal of a central transport mechanism is expected to have a profound impact on the cellular state or function. In theory, the cells may adapt to the loss of transport activity by compensatory increases in the remaining or alternative transport pathways or the cells might switch to a more dormant state. In the CP, Ncbe is likely the major Na^+^ import mechanism to sustain transcellular salt and water movement for CSF secretion [2, 12, 26, 27]. Genetic disruption of Ncbe resulted in a reduced volume of the brain ventricles, which was paralleled by the decrease in the Na^+^-dependent HCO_3_^−^ uptake in CPECs [12]. We reported that the abundance and membrane targeting of various proteins in the CPECs were affected by Ncbe ko [13, 15]. Here, we provide a comprehensive overview of the observed cellular differences within the CP following Ncbe deletion. We provide a novel model for similar assessment in other transporting epithelia during disrupted Na^+^ transport.

The mass spectrometry analysis was validated by comparing new and previously published data on plasma membrane transport proteins abundance in the CP from Ncbe ko and Ncbe wt mice obtained by two antibody-based techniques: immunofluorescence histochemistry and immunoblotting. There is a good general agreement among the three techniques, regardless of the fact that mass spectrometry and immunoblotting were performed on the whole CP tissues, whereas immunofluorescence histochemistry provided data specifically from CPECs. However, the performance of immunofluorescence histochemistry was more variable and therefore tends to decrease the correlation coefficient in comparisons with this technique. This seems to be caused by antibody performance rather than the specific epithelial information, as the connective tissue and blood vessels produced only negligible signal by this technique. Go-term analysis of our mass spectrometry data indicated that a large fraction of proteins with altered abundance in Ncbe ko CP compared to Ncbe wt CP are involved in metabolic activity. Closer analysis of the involved metabolic pathways, such as glycolysis, citric acid cycle, and oxidative phosphorylation, strongly suggested that the cellular capacity for ATP synthesis is systematically reduced in Ncbe ko compared to Ncbe wt CP. By contrast, the capacity for glycogen metabolism is likely enhanced. We confirmed altered abundance of the two enzymes with the largest deviations between the two genotypes with respect to metabolism: glycogen phosphorylase and cytochrome C.

Plausibly, the current data indicate two opposite explanatory mechanisms in the Ncbe CPECs; (1) metabolic changes in Ncbe ko CP, such as the decreased capacity for ATP synthesis, induce changes in membrane transporter expression, or (2) decreased ion transport activity changes metabolic activity in CPECs. The first possibility seems to be ruled out by both the elevated ATP level, as well as the pattern of transcriptional regulators of metabolism including PGC-1 and Sirtuin-2 in Ncbe ko CP. The changes in protein abundance for CTCF/GTF II–II could be interpreted as a compensatory reaction to an apparent decreased capacity for respiratory ATP synthesis, while the TUFM level in the CP from Ncbe ko mice is in accordance with a lower level of mitochondrial biogenesis.

The second hypothesis suggests that the lower abundance of mitochondrial enzymes involved in ATP generation in the Ncbe ko CP is a reaction to lower energy consumption and elevated ATP levels—a Warburg effect-like pattern. Several separate lines of evidence point to low transport activity as the primary signal in cellular protein expression. Firstly, CPECs from Ncbe ko mice are smaller than Ncbe wt cells and the tissue K^+^ content is reduced significantly in Ncbe ko cells despite sufficient ATP levels. This is consistent with previous reports of decreased secretion by CP in conditions such as ageing, where decreased cell size, lower K^+^ contents, elevated ATP levels are also observed [28, 29]. Secondly, proteins from two major systems regulating ion transport function have changed protein abundance in Ncbe ko CPECs compared to Ncbe wt CPECs: IRBIT and OSR1. IRBIT is a Ca^2+^/CaMKII sensitive competitive inhibitor of the IP_3_ receptor involved in enhancing epithelial HCO_3_^−^ transport through activation of mechanisms such as Na^+^-coupled HCO_3_^−^ transporters NBCe1, NBCn1, and NDCBE, as well as NHE3, CFTR and *slc26* derived ion transporters [24]. We found that IRBIT colocalizes with Ncbe in the basolateral membrane domain of the CPECs, that Ncbe co-immunoprecipitates IRBIT from the CP, and that close association between the two proteins in CPECs could be confirmed by PLA assays. However, the regulation of Ncbe by IRBIT has not yet been investigated directly. NBCn1 is, like Ncbe and Ae2, expressed in the basolateral membrane domain in these cells. However, it is not thought to be involved in vectorial transport in this epithelium, as it is a luminal membrane protein in some rodents and areas of the human CP [14, 27, 30]. Thus, NBCn1 is not likely to be the main target for IRBIT in the CP epithelium. In Ncbe ko CP, IRBIT abundance is higher than in Ncbe wt, indicating an attempt to compensate for lower HCO_3_^−^ transport capacity in the Ncbe ko. Indeed, this may result in the observed enhancement in Ae2 expression in Ncbe ko CP, as this transporter belongs to the same IRBIT regulated gene family of HCO_3_^−^ transporters as NBCe1. As judged from the lower secretory activity (small cell volume, high ATP level, low K^+^ level) and lower transport capacity of key ion transporters and the AQP1, the induction of basolateral Ae2 expression (Cl^−^ import) does not seem to compensate for Ncbe deletion in the secretory pathway.

The WNK-SPAK/OSR1 pathway regulates the functional activity of an array of transporters involved in epithelial absorption and secretion, such as NCC, NKCC1, ROMK, NBCe1, CFTR and *slc26* derived anion transporters [24]. It may seem contradictory that the general OSR1 expression is higher in Ncbe ko CP than in the Ncbe wt tissue, while the phosphorylated (activated) form of OSR1 is lower in Ncbe ko. However, this also indicates a divergence of long-term regulation of OSR1 expression and short-term OSR1 regulation by phosphorylation. The resulting overall effect on OSR1 by genetic disruption of Ncbe seems to be a reduced activity. It may seem contradictory that Ncbe co-immunoprecipitated SPAK. Opposite Ncbe, the majority of SPAK is localized to luminal cell domains. However, minor amounts of SPAK may be distributed throughout the cytoplasm, as it is not membrane associated. This is actually indicated by immunofluorescence microscopy on higher intensity exposure (not shown). Also, protein interactions in co-IP assays might form after tissue homogenization and represent an artefact. Again, further PLA should be performed to solve this question.

The lack of Na^+^ import by Ncbe in the ko model reduces cell size and decreases the influx of Na^+^ for luminal extrusion by the Na^+^,K^+^-ATPase, NBCe2 and perhaps NKCC1. In turn, a lower pumping activity would lead to elevation of intracellular K^+^ and lower ATP consumption. A reduction in cell size would normally activate WNK (increased phosphorylation due to reduced intracellular chloride), that should activate SPAK/OSR1 [24]. We did not detect WNKs in the current study, but have previously reported the expression of WNK1 in the CP [31].

The Na^+^,K^+^-ATPase subunits α1, β1, and the accessory subunit phospholemman are the dominant subunits of the luminal pump complex in the CPECs [18]. These Na^+^,K^+^-ATPase subunits are among the proteins of lowest relative abundance in the Ncbe ko tissue by mass spectrometry analysis and thus more affected than other key transporters in the secretory process. Increased Na^+^-influx, cell swelling, [Na^+^]_i_ increase, [K^+^]_i_ decrease normally increases Na^+^,K^+^-ATPase transcription, surface abundance, and activity levels [32, 33]. While Ncbe ko CPECs appear to experience lower Na^+^-influx, cell shrinkage would tend to restrict Na^+^,K^+^-ATPase expression and activity, whereas the reduced intracellular K^+^ content would have the opposite effect. Thus, it would appear that the Na^+^ influx per se or the cell shrinkage are the main mediators of reduced Na^+^,K^+^-ATPase expression in Ncbe ko CPECs. This is in accordance with other models of reduced transport by CPECs and cell size [8, 34, 35]. A mirror phenomenon was observed in cultured renal epithelial cells, where enhanced Na^+^ influx increased Na^+^,K^+^-ATPase surface expression [36], indicating that posttranslational regulation exists in addition to the transcriptional control of the Na^+^,K^+^-ATPase. We hypothesize that the [Na^+^]_i_/K^+^]_i_ is the trigger for the reduced Na^+^,K^+^-ATPase expression in the CPECs from Ncbe ko mice.

The general pattern of differences in protein expression in Ncbe ko vs. Ncbe wt bears some resemblance to the core findings from transcriptomic and proteomic studies of conditions with putatively reduced cerebrospinal fluid secretion such as ageing and Alzheimer’s disease. Na^+^,K^+^-ATPase and AQP1 expression levels are decreased in the CP from ageing rats [28], and the CP in ageing sheep has a reduced Na^+^ uptake, high ATP content, and relative mitochondrial deficiency in addition to a low CSF secretion rate [29]. Similar to the current study, a data mining study of Alzheimer’s disease data demonstrated a downregulated expression of NKCC1, Ncbe, Na^+^,K^+^-ATPase subunits (except for the α1 subunit), and ATP synthase subunits in the CP [37]. A transcriptome study of human Alzheimer’s disease CP material documented a reduced expression of the corresponding transcripts [38]. It is interesting, that many of the changes observed in ageing and Alzheimer’s disease resemble the Ncbe ko model with respect to these central features of the CP epithelium.

## Conclusion

Disruption of Ncbe induces several alterations in cellular metabolism such as glucose, glycogen, fatty acid and amino acid metabolism, and redox reactions. Notably, there is a systematic decrease in capacity for mitochondrial ATP synthesis, mitochondrial area and cytochrome C expression. Nevertheless, the cellular ATP concentration was robustly elevated suggesting that the changes in transport capacity is not secondary to lack of cellular energy supply. The CPECs are smaller in the Ncbe ko model and the cellular K^+^ contents lower, which is consistent with a reduced Na^+^ entry and Na^+^,K^+^-ATPase activity in the cells. Thus, the loss of the putative main basolateral Na^+^-loader Ncbe seems to shift the epithelial cells from an efficient transporting program to an almost dormant program resembling aging and Alzheimer’s disease.

## Supplementary information

**Additional file 1: Figure S1.** Semi-quantitation of immunofluorescence images. (A) The original image stained for cytochrome C. (B) The same image including a manual region of interest (in green). (C) A binary mask of the fluorescence signal above threshold within the region of interest. (D) The resulting image of the minimum values for each pixel in the original image and the mask (i.e. the fluorescence signal above threshold within the region of interest). (E) The corresponding image of the nuclear fluorescence stain and outline of counted nuclei.

**Additional file 2: Figure S2.** Pairwise comparison of the 20 proteins semi-quantified by 2 of the 3 techniques applied in the study. (A) Plot depicting the protein abundance ratios (Ncbe wt/Ncbe ko) obtained by immunoblotting against mass spectrometry. (B) Similar plot of protein abundance ratios assessed by immunofluorescence against mass spectrometry. (C) Similar plot comparing the protein abundance ratios between immunofluorescence and immunoblotting techniques. Dotted lines are lines of perfect concordance; continuous lines represent best-fitted linear regression (Mean ± SEM, n = 5).

**Additional file 3: Figure S3.** Scatter plots showing the relative changes in abundance between Ncbe wt (black bars) and Ncbe ko (grey bars) CP among proteins involved in (A) glycolysis, (B) glycogen, (C) fatty acid and (D) amino acid metabolism as determined by quantitative mass spectrometry (* p < 0.05, X: Failed FDR of 1%, n = 5). Mean values are normalized to control (Ncbe wt) and indicated by horizontal bars. Black triangles indicate data points from Ncbe wt CP, whereas gray circles represent data from Ncbe ko CP.

**Additional file 4: Figure S4.** Scatter plot showing the relative changes in abundance between Ncbe wt (black bars) and Ncbe ko (grey bars) CP among proteins involved in the tricarboxylic acid (TCA) cycle as determined by quantitative mass spectrometry (* p < 0.05, X: Failed FDR of 1%, n = 5). Mean values are normalized to control (Ncbe wt) and indicated by horizontal bars. Black triangles indicate data points from Ncbe wt CP, whereas gray circles represent data from Ncbe ko CP.

**Additional file 5: Figure S5.** Scatter plots showing the relative changes in abundance between Ncbe wt (black bars) and Ncbe ko (grey bars) CP among proteins involved in oxidative phosphorylation: (A) Complex I of the respiratory chain, (B) Complexes II, III, and IV of the respiratory chain as determined by quantitative mass spectrometry (* p < 0.05, X: Failed FDR of 1%, n = 5). Mean values are normalized to control (Ncbe wt) and indicated by horizontal bars. Black triangles indicate data points from Ncbe wt CP, whereas gray circles represent data from Ncbe ko CP.

**Additional file 6: Figure S6.** Scatter plots showing the relative changes in abundance between Ncbe wt (black bars) and Ncbe ko (grey bars) ko CP among proteins involved in (A) mitochondrial ATP synthesis, (B) mitochondrial transport, and (C) redox reactions as determined by quantitative mass spectrometry (* p < 0.05, X: Failed FDR of 1%, n = 5). Mean values are normalized to control (Ncbe wt) and indicated by horizontal bars. Black triangles indicate data points from Ncbe wt CP, whereas gray circles represent data from Ncbe ko CP.

**Additional file 7: Table S1.** Proteins identified by co-immunoprecipitation with anti-Ncbe antibody as bait.

## Data Availability

The datasets generated during and/or analysed during the current study are available in the Interpret repository, http://interpretdb.au.dk/database/CPE_TMT/CPE_TMT_proteome.html.

## Abbreviations

Ae2: anion exchanger 2
AMP: adenosine monophosphate
AMPK: AMP-activated protein kinase
AQP1: aquaporin 1
ATP: adenosine triphosphate
BH: Benjamini–Hochberg
BSA: bovine serum albumin
Ceacam-2: carcino-embryonic antigen-related cell adhesion molecule 2
CP: choroid plexus
CPECs: choroid plexus epithelial cells
CSF: cerebrospinal fluid
CTCF: CCCTC-binding factor
EDTA: ethylene diamine-tetraacetic acid
EGTA: ethylene glycol-bis(β-aminoethyl ether)-tetraacetic acid
FACS: fluorescence assisted cell sorting
FDR: false discovery rate
GO: gene ontology
GTF II-I: general transcription factor II-I
HBS: Hepes-buffered salt solution
IRBIT: IP3R binding protein released with inositol 1,4,5-trisphosphate
ko: knockout
MS: mass spectrometry
MS/MS: tandem mass spectrometry
NBCe2: electrogenic Na^+^:HCO_3_^−^ transporter 2
Ncbe: sodium-dependent chloride bicarbonate exchanger
NCE: normalized collision energy
NKCC1: Na^+^,K^+^,2Cl^−^ cotransporter 1
nLC: nano liquid chromatography
OSR1: oxidative stress-responsive kinase 1
PANTHER: protein analysis through evolutionary relationships
PBS: phosphate buffered salt solution
PGC-1α: peroxisome proliferator-activated receptor γ coactivator 1α
PLA: proximity ligation assay
PVDF: polyvinylidene difluoride
Slc4a10: solute carrier family 4 member 10
SPAK: SPS1-related Proline/Alanine-rich Kinase
TEG: tris-EGTA
TMT: tandem mass tag
TUFM: mitochondrial Tu translation elongation factor
WNK: with-no lysine (K)
wt: wildtype
