# Supplementary figures and images for: Genetic disruption of slc4a10 alters the capacity for cellular metabolism and vectorial ion transport in the choroid plexus epithelium
Source: Fluids Barriers CNS. 2020 Jan 7;17:2. doi: 10.1186/s12987-019-0162-5 (PMC6945596; doi:10.1186/s12987-019-0162-5)

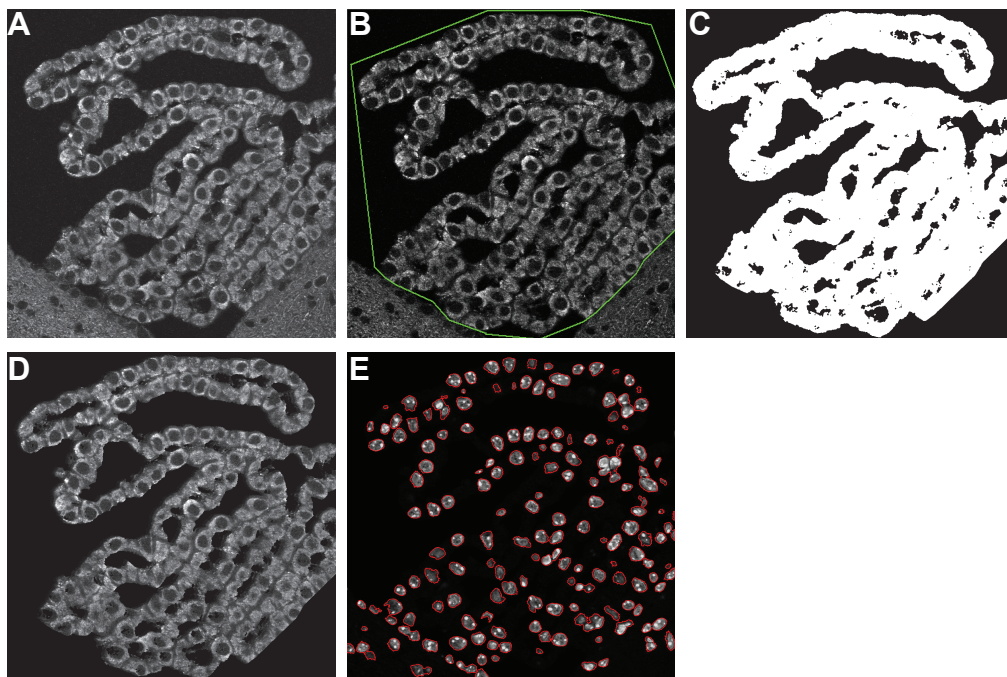

Supplemental Figure 1

Supplement: Supplementary file 1 — Additional file 1: Figure S1. Semi-quantitation of immunofluorescence images. (A) The original image stained for cytochrome C. (B) The same image including a manual region of interest (in green). (C) A binary mask of the fluorescence signal above threshold within the region of interest. (D) The resulting image of the minimum values for each pixel in the original image and the mask (i.e. the fluorescence signal above threshold within the region of interest). (E) The corresponding image of the nuclear fluorescence stain and outline of counted nuclei. [file 12987_2019_162_MOESM1_ESM.pdf]

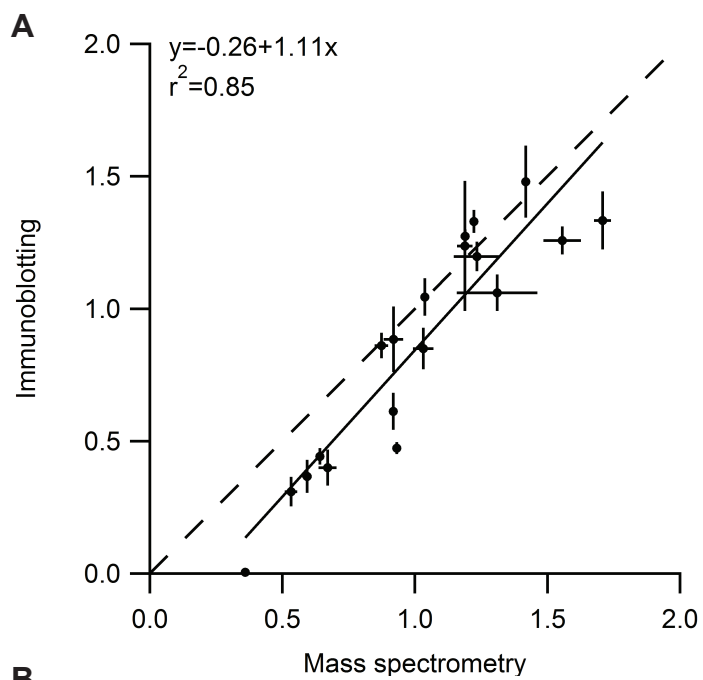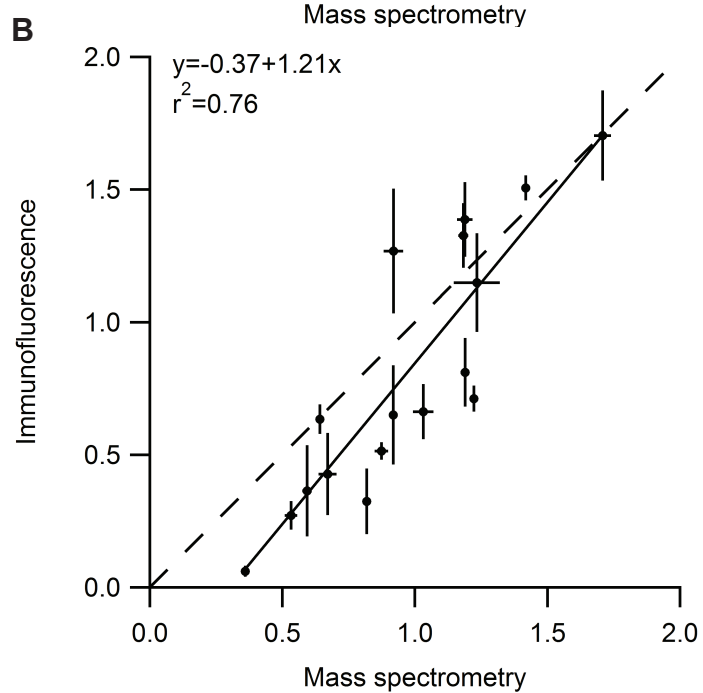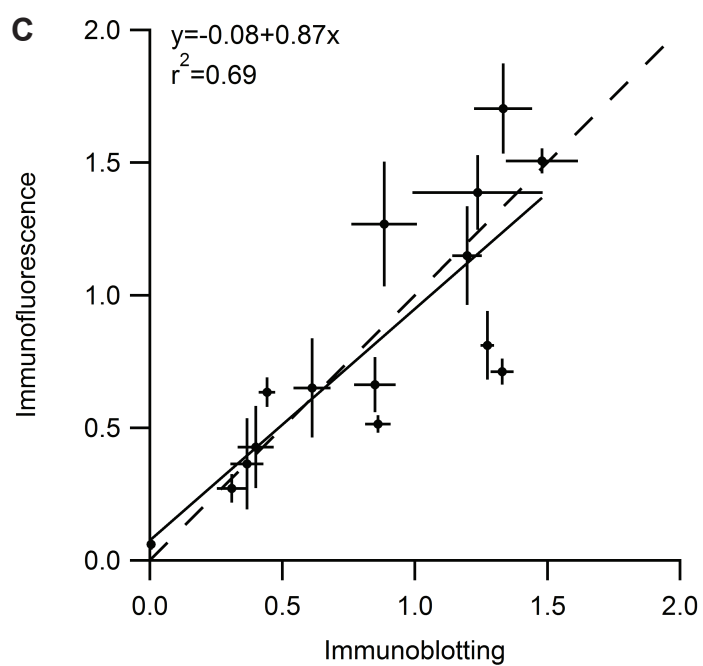

Supplemental Figure 2

Supplement: Supplementary file 2 — Additional file 2: Figure S2. Pairwise comparison of the 20 proteins semi-quantified by 2 of the 3 techniques applied in the study. (A) Plot depicting the protein abundance ratios (Ncbe wt/Ncbe ko) obtained by immunoblotting against mass spectrometry. (B) Similar plot of protein abundance ratios assessed by immunofluorescence against mass spectrometry. (C) Similar plot comparing the protein abundance ratios between immunofluorescence and immunoblotting techniques. Dotted lines are lines of perfect concordance; continuous lines represent best-fitted linear regression (Mean ± SEM, n = 5). [file 12987_2019_162_MOESM2_ESM.pdf]

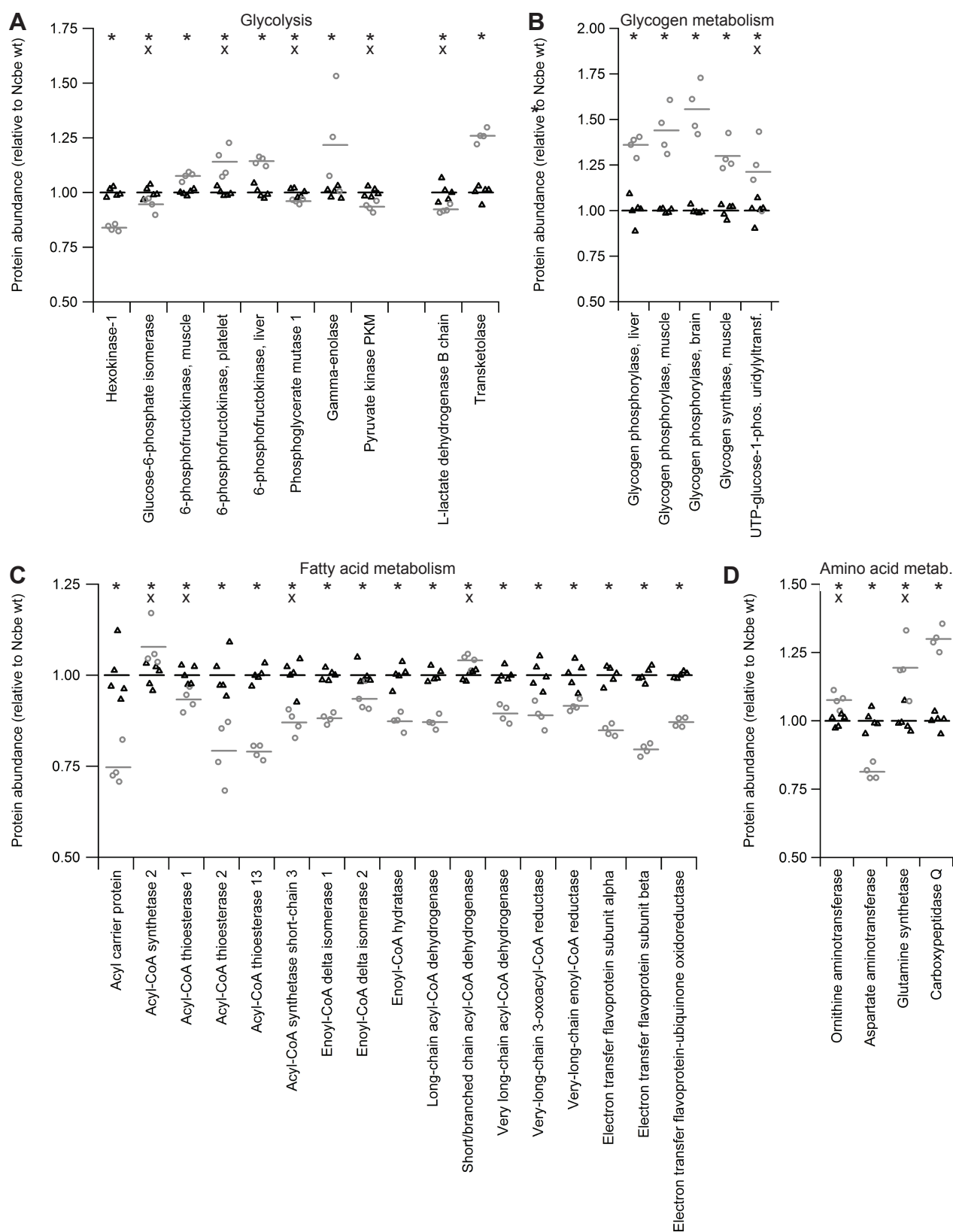

Supplemental Figure 3

Supplement: Supplementary file 3 — Additional file 3: Figure S3. Scatter plots showing the relative changes in abundance between Ncbe wt (black bars) and Ncbe ko (grey bars) CP among proteins involved in (A) glycolysis, (B) glycogen, (C) fatty acid and (D) amino acid metabolism as determined by quantitative mass spectrometry (* p < 0.05, X: Failed FDR of 1%, n = 5). Mean values are normalized to control (Ncbe wt) and indicated by horizontal bars. Black triangles indicate data points from Ncbe wt CP, whereas gray circles represent data from Ncbe ko CP. [file 12987_2019_162_MOESM3_ESM.pdf]

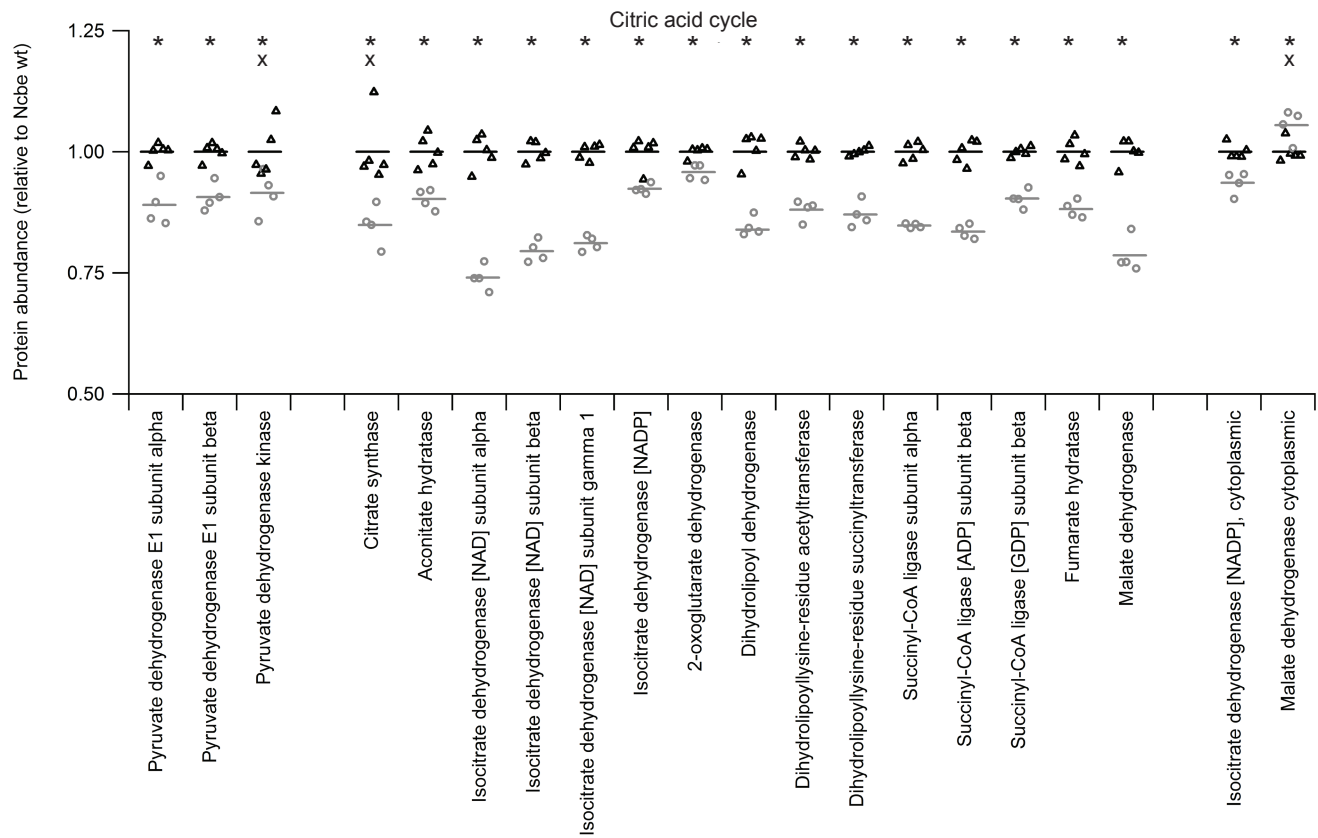

Supplemental Figure 4

Supplement: Supplementary file 4 — Additional file 4: Figure S4. Scatter plot showing the relative changes in abundance between Ncbe wt (black bars) and Ncbe ko (grey bars) CP among proteins involved in the tricarboxylic acid (TCA) cycle as determined by quantitative mass spectrometry (* p < 0.05, X: Failed FDR of 1%, n = 5). Mean values are normalized to control (Ncbe wt) and indicated by horizontal bars. Black triangles indicate data points from Ncbe wt CP, whereas gray circles represent data from Ncbe ko CP. [file 12987_2019_162_MOESM4_ESM.pdf]

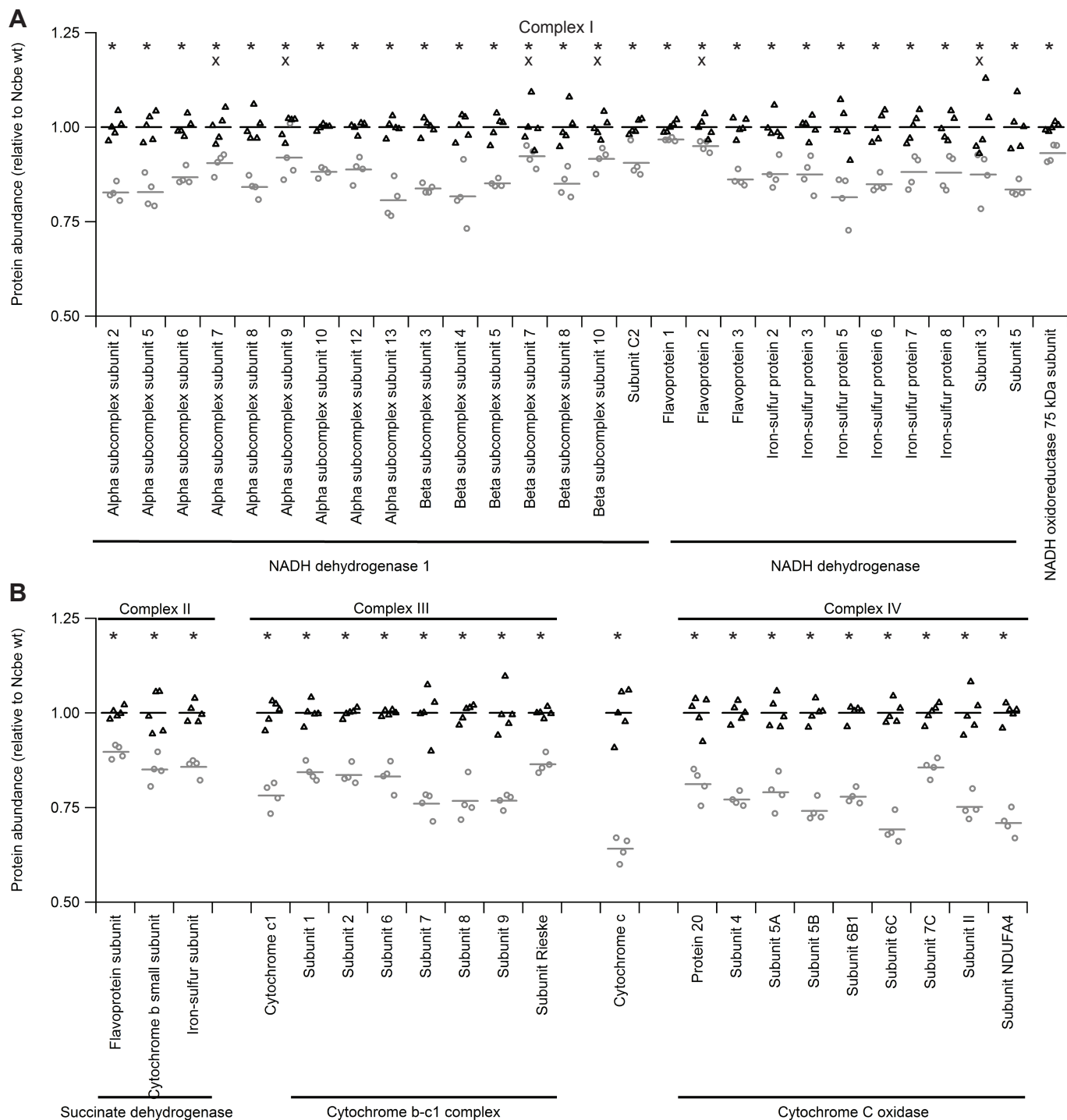

Supplemental Figure 5

Supplement: Supplementary file 5 — Additional file 5: Figure S5. Scatter plots showing the relative changes in abundance between Ncbe wt (black bars) and Ncbe ko (grey bars) CP among proteins involved in oxidative phosphorylation: (A) Complex I of the respiratory chain, (B) Complexes II, III, and IV of the respiratory chain as determined by quantitative mass spectrometry (* p < 0.05, X: Failed FDR of 1%, n = 5). Mean values are normalized to control (Ncbe wt) and indicated by horizontal bars. Black triangles indicate data points from Ncbe wt CP, whereas gray circles represent data from Ncbe ko CP. [file 12987_2019_162_MOESM5_ESM.pdf]

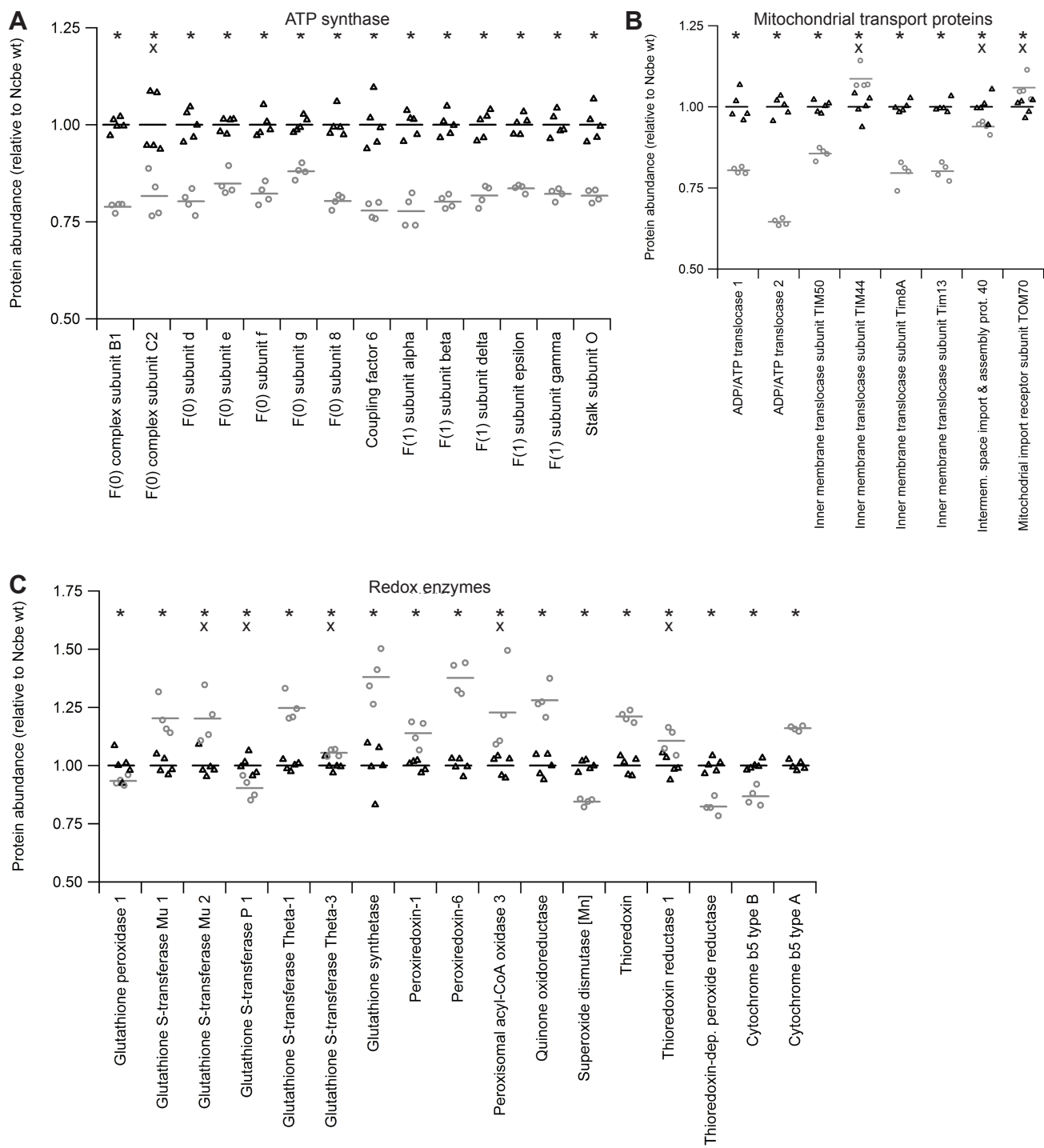

Supplemental Figure 6

Supplement: Supplementary file 6 — Additional file 6: Figure S6. Scatter plots showing the relative changes in abundance between Ncbe wt (black bars) and Ncbe ko (grey bars) ko CP among proteins involved in (A) mitochondrial ATP synthesis, (B) mitochondrial transport, and (C) redox reactions as determined by quantitative mass spectrometry (* p < 0.05, X: Failed FDR of 1%, n = 5). Mean values are normalized to control (Ncbe wt) and indicated by horizontal bars. Black triangles indicate data points from Ncbe wt CP, whereas gray circles represent data from Ncbe ko CP. [file 12987_2019_162_MOESM6_ESM.pdf]
