# Supplementary material for: Genetic disruption of slc4a10 alters the capacity for cellular metabolism and vectorial ion transport in the choroid plexus epithelium
Source: Fluids Barriers CNS. 2020 Jan 7;17:2. doi: 10.1186/s12987-019-0162-5 (PMC6945596; doi:10.1186/s12987-019-0162-5)
Supplement: Supplementary file 7 — Additional file 7: Table S1. Proteins identified by co-immunoprecipitation with anti-Ncbe antibody as bait. [file 12987_2019_162_MOESM7_ESM.docx]

**Table S1.** Proteins identified by co-immunoprecipitation with anti-Ncbe antibody as bait.

| Accession | Description | Name | App |
| --- | --- | --- | --- |
| 14192922 | actin, alpha cardiac muscle 1 | α-actin | 3 |
| 568933073 | anion exchange protein 2 X4 | Ae2 | 2 |
| 568955180 | AP-1 complex subunit mu-1 X1 | AP-1 µ-1 | 2 |
| 569011644 | AP-1 complex subunit sigma-2 X2 | AP-1 σ-2 | 2 |
| 163644277 | AP-2 complex subunit alpha-2 | AP-2 α-2 | 2 |
| 163310776 | AP-3 complex subunit beta-1 | AP-3 β-1 | 2 |
| 170763481 | AP-3 complex subunit mu-2 | AP-3 µ-2 | 2 |
| 6680710 | aquaporin-1 | AQP1 | 2 |
| 568908679 | band 4.1-like protein 5 X3 | band 4.1-like 5 | 2 |
| 568991833 | beta-1-syntrophin X1 | β1-syntrophin | 2 |
| 6678059 | beta-2-syntrophin | β2-syntrophin | 2 |
| 22550094 | cAMP-dependent protein kinase type II-alpha regul. | PKA II-α | 2 |
| 755509715 | protein 4.1 X21 | protein 4.1 | 2 |
| 283837832 | putative adenosylhomocysteinase 3, 3 | IRBIT | 2 |
| 176866245 | sodium-driven chloride bicarbonate exchanger 2 | Ncbe 2 | 3 |
| 334688858 | sodium-driven chloride bicarbonate exchanger 5 | Ncbe 5 | 3 |
| 569003445 | solute carrier family 12 member 2 X1 | NKCC1 | 2 |
| 569005698 | spectrin beta chain, non-erythrocytic 2 X1 | β2-spectrin | 2 |
| 568917807 | STE20/SPS1-rel. proline-alanine-rich protein kinase | SPAK | 2 |
| 31543940 | vesicle-assoc. membr. protein-associated protein B | Vamp33b | 2 |

Results are the proteins identified by MS repeatedly and specific to anti-Ncbe (compared to proteasome 20s).
